# A Multiresponsive Ferrocene‐Based Chiral Overcrowded Alkene Twisting Liquid Crystals

**DOI:** 10.1002/anie.202413047

**Published:** 2024-11-06

**Authors:** Maximilian Fellert, Robert Hein, Alexander Ryabchun, Yohan Gisbert, Charlotte N. Stindt, Ben L. Feringa

**Affiliations:** ^1^ Stratingh Institute for Chemistry University of Groningen Nijenborgh 3 9747 AG Groningen The Netherlands

**Keywords:** Liquid crystals, Redox chemistry, Ferrocene, Molecular Switch, Asymmetric synthesis

## Abstract

The reversible modulation of chirality has gained significant attention not only for fundamental stereochemical studies but also for numerous applications ranging from liquid crystals (LCs) to molecular motors and machines. This requires the construction of switchable molecules with (multiple) chiral elements in a highly enantioselective manner, which is often a significant synthetic challenge. Here, we show that the dimerization of an easily accessible enantiopure planar chiral ferrocene‐indanone building block affords a multi‐stimuli‐responsive dimer (FcD) with pre‐determined double bond geometry, helical chirality, and relative orientation of the two ferrocene motifs in high yield. This intrinsically planar chiral switch can not only undergo thermal or photochemical *E/Z* isomerization but can also be reversibly and quantitatively oxidized to both a monocationic and a dicationic state which is associated with significant changes in its (chir)optical properties. Specifically, FcD acts as a chiral dopant for cholesteric LCs with a helical twisting power (HTP) of 13 μm^−1^ which, upon oxidation, drops to near zero, resulting in an unprecedently large redox‐tuning of the LC reflection color by up to 84 nm. Due to the straightforward stereoselective synthesis, FcD, and related chiral switches, are envisioned to be powerful building blocks for multi‐stimuli‐responsive molecular machines and in LC‐based materials.

## Graphical abstract

We present a multi‐stimuli‐responsive dimer (FcD) based on an enantiopure planar chiral ferrocene‐indanone. FcD exhibits thermal/photochemical E/Z isomerization and is redox‐switchable, which significantly alters its chiral expression and optical properties. As a chiral dopant in cholesteric liquid crystals, FcD enables redox‐tuning of the liquid crystal reflection color by up to 84 nm, showcasing its potential in functional materials.

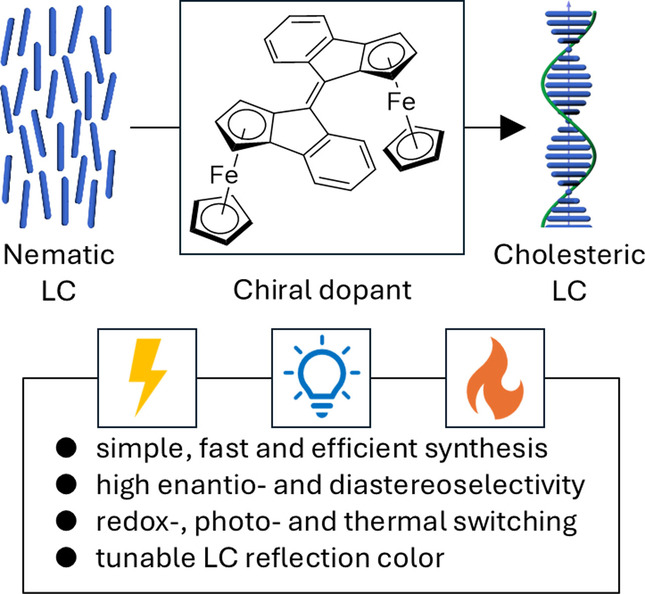

## Introduction

Over the past two decades, scientists have miniaturized a toolbox of macroscopic devices to the nanoscale, creating molecular switches,[^1^, ^2^, ^3^, ^4^] motors,[^5^, ^6^, ^7^, ^8^, ^9^, ^10^, ^11^] and machines[^12^, ^13^, ^14^, ^15^, ^16^, ^17^, ^18^, ^19^, ^20^] and enabling mechanical motion in, among others, artificial muscles,[^21^, ^22^, ^23^, ^24^] pumps,[^25^, ^26^, ^27^, ^28^] channels,[^29^, ^30^] transporters,[^31^, ^32^] and shuttles.[^33^, ^34^] To bring switches to the molecular level, overcrowded alkenes have played a significant role, and due to their unique addressability by photo‐, thermo‐, and electrochemical stimuli, they have been utilized to enable, inter alia, control of motion,[^35^, ^36^, ^37^] responsive transition metal complexes,^[38]^ and optical data storage.^[39]^ Steric hindrance around the central double bond of these alkenes causes a deviation from planarity, thereby inducing the formation of helices and thus stereoisomers. In the presence of additional point chirality, the helical chirality of these switches can be controlled, which enabled the design of unidirectional molecular motors which, since their discovery,^[40]^ have been intensively studied and implemented in various fields of chemistry,[^41^, ^42^] such as drug delivery,[^43^, ^44^] MOFs and COFs,[^45^, ^46^, ^47^, ^48^] surfaces,[^49^, ^50^] liquid crystals (LCs),[^51^, ^52^, ^53^, ^54^] coupled rotors,[^55^, ^56^] adaptive catalysts,[^57^, ^58^] and smart materials.[^59^, ^60^, ^61^]

Access to enantiopure overcrowded alkenes[^62^, ^63^, ^64^] is crucial for many of their applications and various methods have been developed, based on separation by chiral chromatography,[^65^, ^66^] direct asymmetric synthesis,[^67^, ^68^, ^69^, ^70^, ^71^, ^72^, ^73^] and the use of chiral auxiliaries.^[74]^ However, these methods often show limitations such as low yields, reduced scale, or restriction to specifically functionalized molecules. Hence, novel approaches to construct enantiopure overcrowded alkenes are highly sought‐after. A potentially powerful, but largely unexplored path towards novel chiral switches is the use of planar chiral building blocks, such as paracyclophanes, rigid cycloalkenes, bridged annulenes or di‐substituted ferrocenes, whose (asymmetric) synthesis has recently gained considerable attention.^[75]^ We envisioned that in addition to their chirality, the redox activity of ferrocenes renders them particularly unique building blocks for the construction of novel multi‐stimuli‐responsive systems based on overcrowded alkenes.

A potential application of such chiral, redox‐active switches is their use as dopants to convert a nematic (LC) phase into a cholesteric phase where the molecular chirality of the dopant is transduced to the LC environment, yielding a helical supramolecular architecture. The periodic character of supramolecular cholesteric helices, described by the helix pitch (*p*), causes a number of unique optical properties[^76^, ^77^] finding a broad range of applications beyond display technologies in fields such as tunable optics, photonics, lasers and sensors.[^78^, ^79^] Significant attention has been directed at stimuli‐responsive chiral dopants, in particular photoswitchable ones, which are capable of significantly changing the optical properties of the cholesteric materials by photoisomerization[^80^, ^81^, ^82^, ^83^, ^84^] or photocyclization.[^85^, ^86^] An alternative to light‐responsive chiral dopants, which are so far largely represented by azobenzene derivatives in combination with chiral additives and overcrowded‐alkene‐based molecular motors,[^87^, ^88^, ^89^, ^90^] can be redox‐active intrinsically chiral molecules that can modulate the helical twisting power (HTP), and hence the properties of the cholesteric material, upon oxidation or reduction. Despite the appeal of this approach, there are only a few reports on such systems used in LCs.[^91^, ^92^, ^93^] In their pioneering work, Aida and co‐workers developed a dopant consisting of an axially chiral BINOL fragment with ferrocene attached via a flexible aliphatic spacer. However, this dopant design has some fundamental limitations in the ability to change the pitch of the cholesteric helix due to the fact that the chiral moiety and the redox unit of the molecule are spaced apart, such that the electrochemical switching has no strong effect on the chiral expression of the BINOL fragment.^[91]^ Conceivably, we expect that a more intimate coupling of redox‐active and chiral motifs using an inherently chiral redox system would enable more significant modulation of the HTP via redox stimuli.

To this end, we present an efficient, high yielding, enantio‐ and stereoselective synthesis of the redox‐active, chiral molecular switch FcD based on a planar chiral ferrocene building block. Detailed electrochemical, thermal, and photophysical studies, assisted by DFT calculations, revealed rich and highly reversible switching behavior by heat, light or redox stimulation, which is associated with significant changes in the switches’ geometry and chiral expression. The use of this multi‐stimuli‐responsive switch as a redox‐responsive chiral dopant for nematic LC phases enables unprecedentedly large, and reversible changes in the HTP and thus reflection colors of the LC materials.

## Results and Discussion

### Synthesis and Structural Analysis

**Figure 1:**
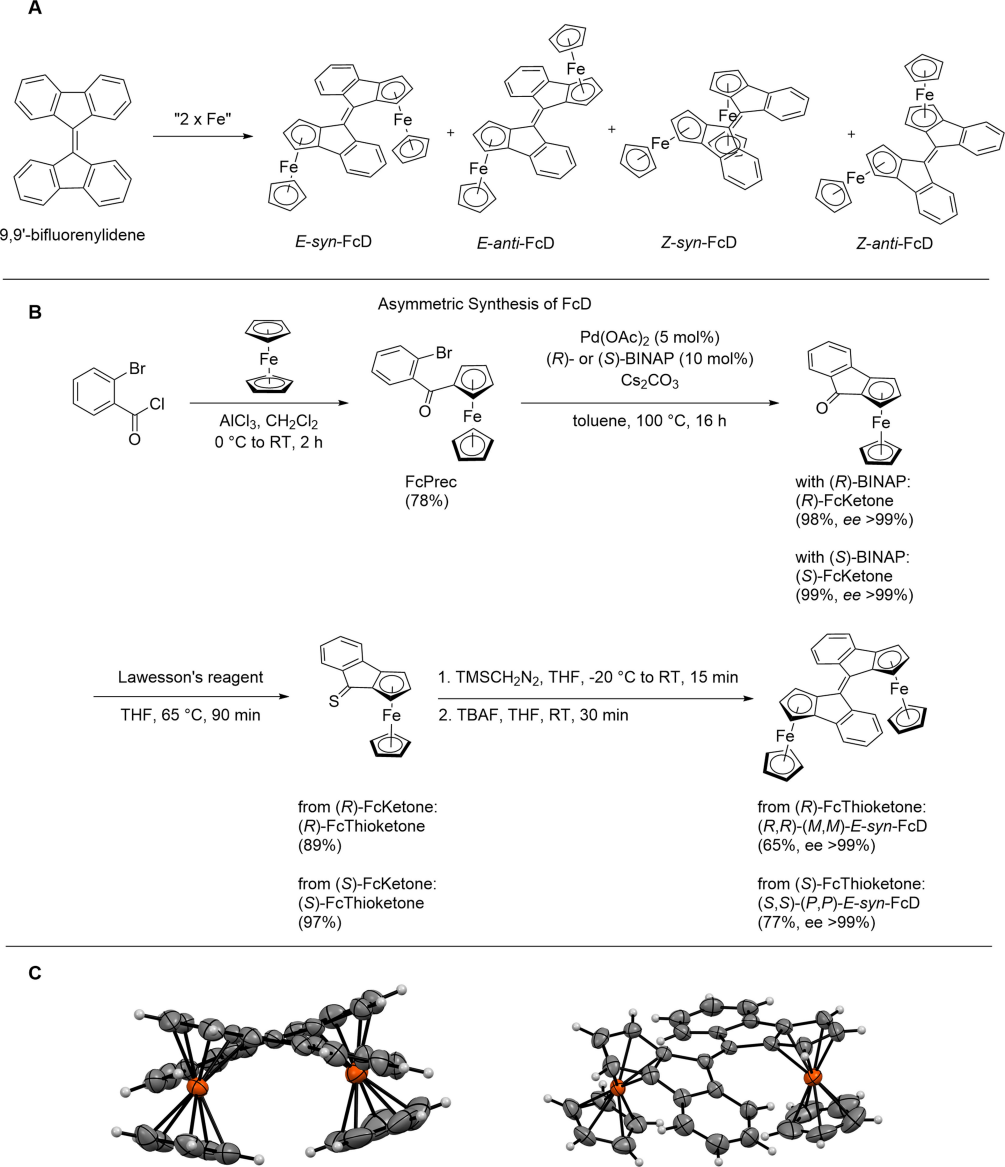
A: Possible *E*‐*syn*, *E*‐*anti*, Z‐*syn*, and *Z*‐*anti* isomers of FcD, obtained by integration of two ferrocene units into the archetypical 9,9′‐bifluorenylidene overcrowded alkene switch scaffold, B: Asymmetric synthesis of FcD. For clarity, only the products with (*R*)‐ and (*R*,*R*)‐configuration are shown, C: Side and top view of the X‐ray structure of *E*‐*syn*‐FcD, highlighting the twisted orientation of both halves with respect to each other and visualizing the *syn* orientation of the ferrocene moieties as well as the dihedral angle of both helices, respectively.

The integration of a planar chiral ferrocene motif into each half of an overcrowded alkene switch scaffold, such as the archetypical 9,9′‐bifluorenylidene,^[94]^ to afford the FcD dimer can, in principle, result in the formation of a large range of different isomers (Figure 1A). This includes *E/Z* isomers as well as isomers with a different relative orientation of the Fc motif (*syn/anti*) resulting in four unique switch scaffolds (Figure 1A) as well as their enantiomers (not shown). Indeed, simple dimerization of the racemic indanone building block FcKetone results in the formation of a mixture of all four different isomers, which significantly restricts their application scope.^[95]^

Based on our longstanding efforts towards new chiral overcrowded alkenes, we envisioned an asymmetric synthesis of FcD (Figure 1B). First, the commercially available 2‐bromobenzoyl chloride was transformed into the precursor FcPrec by a Friedel–Crafts reaction with ferrocene in 78 % yield. Subsequent Pd‐catalyzed intramolecular coupling following the procedures of You and co‐workers^[96]^ and Gu and co‐workers^[97]^ allowed access to (*R*)‐FcKetone and (*S*)‐FcKetone with excellent yield (98 % and 99 %, respectively) and enantiopurity (both *ee* >99 %). Conversion of (*R*)‐FcKetone and (*S*)‐FcKetone by reaction with Lawesson's reagent led to the formation of (*R*)‐FcThioketone and (*S*)‐FcThioketone in almost quantitative yield (89 % and 97 %, respectively). Finally, (*R*,*R*)‐FcD and (*S*,*S*)‐FcD were synthesized by dimerization of (*R*)‐FcThioketone or (*S*)‐FcThioketone in isolated yields of 65 % and 77 %, respectively, using a procedure previously employed by our group for the synthesis of bisthioxanthylidenes.[^98^, ^99^, ^100^] Gratifyingly, the dimerization proceeds with full diastereoselectivity via a 1,3‐diploar cycloaddition including a five‐membered ring dithiane intermediate. This highly congested intermediate is formed by a cofacial approach of two thioketone halves which is much more sterically favored when the two bulky ferrocene moieties are on opposite sides of the central double bond and are facing away from each other. Collapse of this intermediate to the final alkene switch leads to the exclusive formation of (*R*,*R*)*‐E‐syn*‐FcD and (*S*,*S)*‐*E‐syn*‐FcD, respectively, as the approach of the second ferrocene thioketone in the cycloaddition to obtain the *Z*‐*anti* FcD product would be sterically strongly disfavored. In contrast, the diastereomeric *E‐anti*‐FcD and *Z‐syn*‐FcD would only be accessible by dimerization of (*S*)‐FcThioketone with its enantiomer (*R*)‐FcThioketone.

Single crystals of (*R,R*)‐FcD suitable for X‐ray diffraction (XRD) were obtained by slow evaporation of a concentrated ethyl acetate solution. The XRD analyses further confirmed the absolute configuration of the proposed structure in which the two ferrocene motifs adopt an *E‐syn* orientation (Figure 1C). Moreover, the two bulky ferrocenes point away from each other, thereby inducing a twisting around the central C=C double bond and resulting in two *M* helices with dihedral angles of ~25°. To confirm that this helical isomer is also the most favored conformer in solution, we performed computational studies on (*R*,*R*)‐FcD at the r^2^SCAN‐3c^[101]^ level of theory using the conductor‐like polarizable continuum CPCM(Toluene) solvent model.^[102]^ Indeed, (*R*,*R*)*‐*(*M*,*M*)‐*E*‐*syn*‐FcD was found to be the most stable isomer while the corresponding (*R*,*R*)‐(*P*,*P*)‐*E‐syn‐*FcD, where the two ferrocene motifs point towards each other, thereby resulting in two *P* helices, is 10.2 kJ/mol higher in energy.

The calculated activation energy for this thermal helix inversion (THI) process is very low (ΔG^≠^_THI‐*E‐syn‐*FcD_=37.7 kJ/mol at 25 °C), such that an equilibrium between the (*M,M*) and (*P*,*P*) isomers is instantaneously established (t_1/2_≈0.5 μs at 25 °C) and strongly favors the former isomer (98 : 2 ratio of (*M*,*M*)/(*P*,*P*) for (*R,R*)‐*E*‐*syn*‐FcD at 25 °C).

As a result, the dimerization of the easily accessible enantiopure (*R*)‐ or (*S*)‐FcKetone is associated with the highly selective formation of only one product of defined double bond geometry (*E*), relative orientation of the ferrocene motifs (*syn*) and helicity; (*M*,*M*) for (*R*,*R*)‐FcD and (*P*,*P*) for (*S*,*S*)‐FcD. Remarkably, all three stereochemical elements are defined in a single dimerization step, enabling facile access to these structurally complex inherently chiral molecules. To improve readability, only the most relevant stereodescriptors will be used in the following.

**Figure 2:**
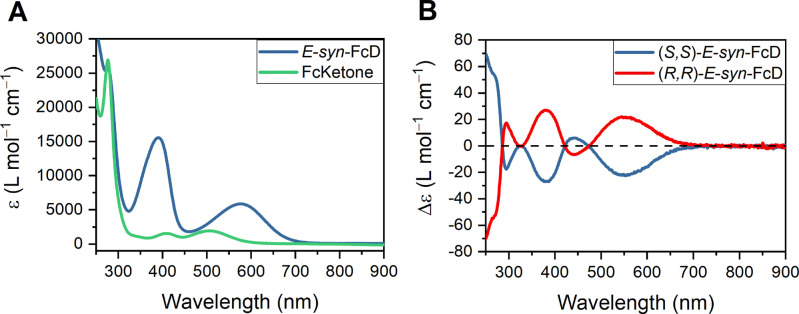
A: UV/Vis spectra of *E‐syn*‐FcD and FcKetone in CH_2_Cl_2_ (50 μM). B: CD spectra of (*S*,*S*)‐*E‐syn*‐FcD and (*R*,*R*)‐*E‐syn*‐FcD in CH_2_Cl_2_ (50 μM).

The UV/Vis spectrum of *E*‐syn‐FcD displays two notable absorption bands at 390 and 576 nm with moderate molar absorption coefficients of ϵ=15580 and 5890 M^−1^ cm^−1^, respectively, in good agreement with the deep purple color of the compound (Figure 2A). These bands are noticeably stronger for FcD in comparison to its precursor FcKetone, whose lowest energy absorbance is centered at 505 nm with a significantly lower molar absorption coefficient of ϵ=1960 M^−1^ cm^−1^. Dimerization is thus associated with a notable red‐shift of absorbance which is indicative of a chromophore that spans the entire molecule through the central, conjugated double bond. Of further note is that both FcKetone and FcD show, by virtue of the ferrocene motif, significantly more red‐shifted absorbance in comparison to their non‐ferrocene containing analogues, such as 9,9′‐bifluorenylidene (λ_max_=458 nm),^[103]^ an important property that is highly sought‐after in the development of visible‐light‐responsive molecular switches and motors.[^2^, ^3^, ^7^, ^11^]

The CD spectra of the enantiomeric (*S*,*S*)‐*E*‐*syn*‐FcD and (*R*,*R*)‐*E*‐*syn*‐FcD are, as expected, mirror images and show notable Cotton bands at 295, 380, 440, and 550 nm with moderate molar extinction coefficients Δϵ of 17, 27, 6 and 22 M^−1^ cm^−1^, respectively (Figure 2B). These spectra are similar to those of the corresponding FcKetone precursors; however, the molar extinction coefficients are significantly larger for the dimers (Figure S19 and S20). We also calculated the absorption dissymmetry factor g_abs_ (g_abs_=Δϵ/ϵ) for *E*‐*syn*‐FcD, revealing a moderate g_abs_ value of ~5×10^−3^ at 525 nm (Figure S21).

### Thermal‐ and Photoswitching

**Figure 3:**
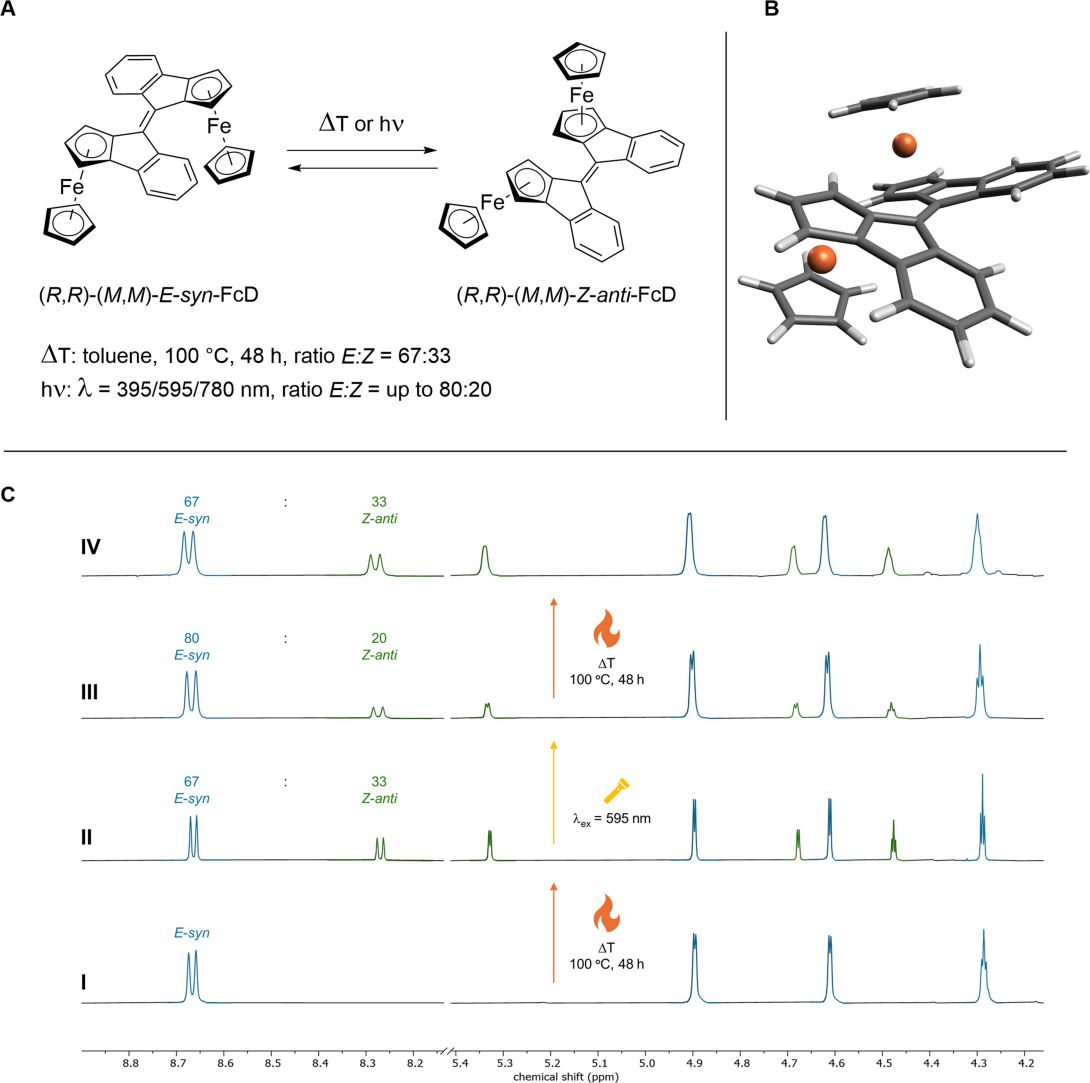
A: Thermal and photoswitching of FcD, B: Calculated structure of (*M*,*M*)‐*Z*‐*anti*‐FcD and C: Switching cycle of *E*‐*syn*‐FcD monitored by ^1^H NMR spectroscopy (600 MHz, toluene‐d_8_, 25 °C: I) Initial sample of *E*‐*syn*‐FcD, II) Mixture obtained after heating at 100 °C for 48 h, III) *E* : *Z* ratio after irradiation with 595 nm light for 10 min at 20 °C and IV) Mixture obtained after a subsequent heating period of 48 h at 100 °C.

FcD is responsive to various external stimuli including heat, which induces thermal *E/Z* isomerization (Figure 3A). Upon heating a sample of *E‐syn*‐FcD to 100 °C, the appearance of *Z‐anti*‐FcD (Figure 3B) was monitored by ^1^H NMR. A thermal equilibrium, resulting in an *E*‐*syn* : *Z‐anti* ratio of 67 : 33 in toluene (Figure 3CII), was reached after approximately two days. This value is in good agreement with the calculated Boltzmann distribution (70 : 30 ratio of *E* and *Z* isomers) at 100 °C (see Table S2). Extending the heating period to seven days in total did not lead to any alteration of the *E* : *Z* ratio. The kinetics of the thermal switching process were then investigated by ^1^H NMR spectroscopy (Figure S13). Due to experimental constraints, the temperature could not be increased above 90 °C, and the heating period was limited to 65 h, after which an *E* : *Z* ratio of 76 : 24 was observed. However, using an exponential fit, the limit value of the *E* : *Z* ratio was estimated to be 69 : 31, which is in accordance with the calculated 71 : 29 Boltzmann distribution at this temperature. Further analysis revealed a half‐life time of t_1/2_=23 h at 90 °C for the thermal switching process.

To further analyze its structure, pure (*R*,*R*)‐*Z*‐*anti*‐FcD was obtained by heating a sample of (*R*,*R*)‐*E*‐*syn*‐FcD in toluene at 100 °C for three days and subsequent separation of the *E*‐*syn*/*Z*‐*anti* mixture by preparative thin layer chromatography (TLC). Due to its sensitivity towards light, which induces rapid backswitching to *E*‐*syn*‐FcD, *Z*‐*anti*‐FcD was characterized by ^1^H and Nuclear Overhauser Effect Spectroscopy (NOESY) NMR spectroscopy. The comparison of the ^1^H NOESY NMR spectra of *E*‐*syn*‐FcD and *Z*‐*anti*‐FcD gives unequivocal insight into their structure and validates the identification of *Z*‐*anti*‐FcD as the thermal and photoswitching product of *E*‐*syn*‐FcD. Specifically, while the proximity of a ferrocene‐bound proton to an aromatic proton in *E*‐*syn*‐FcD causes an observable NOE (Figure S10), the absence of such a signal in the NOESY spectrum of *Z*‐*anti*‐FcD confirms the assignment of its conformation (Figure S11).

The apparent back‐isomerization of *Z*‐*anti*‐FcD by light during its isolation process encouraged us to investigate the photoswitchability of FcD. Among the reported examples of ferrocene‐containing photoswitches^[104]^ such as azobenzenes,[^105^, ^106^] and stilbenes^[107]^ as well as planar chiral pillarenes[^108^, ^109^] and azobenzenes,[^110^, ^111^] FcD emerges as a novel ferrocene‐based planar chiral photoswitch. When irradiated with light at various wavelengths, i.e. 395, 595 or 780 nm, at room temperature, *E*‐*syn*‐FcD is partially transformed into its *Z*‐*anti* isomer, leading to similar *E* : *Z* ratios of *E* : *Z*_395 nm, 20 °C_=80 : 20, *E* : *Z*_595 nm, 20 °C_=82 : 18, and *E* : *Z*_780 nm, 20 °C_=86 : 14, respectively (Figure S16–S18). This highlights the versatility of FcD as a photoswitch, as different wavelengths over the whole range of the visible spectrum of light even up to the near‐infrared regime can be used to initiate the *E*/*Z* isomerization process.

Interestingly, this *E* : *Z* ratio is very close to the calculated Boltzmann distribution of 77 : 23 at 25 °C (see Table S2), suggesting that the photoinduced isomerization did not result in the population of an out‐of‐equilibrium high‐energy state but rather afforded the thermodynamically favored distribution at a temperature where thermal equilibration through *E*/*Z* isomerization is hampered, thus allowing to selectively populate isomeric distributions which are not thermally accessible. To further confirm this hypothesis, the photoisomerization process was studied by in situ variable‐temperature NMR irradiation. Irradiation of a sample of pure (*R*,*R*)‐*E*‐*syn*‐FcD in toluene‐d_8_ with λ=595 nm at −80 °C afforded an *E* : *Z* mixture in a 94 : 6 ratio (see Figure S14), being in line with the calculated Boltzmann distribution of 90 : 10 at the same temperature. Further irradiation of the same sample at 90 °C afforded an *E* : *Z* mixture in a 76 : 23 ratio (see Figure S15), being close to the thermally accessible distribution at this temperature but obtained over a much shorter time of ca. 30 min of irradiation, which is much faster than the aforementioned purely thermal *E*/*Z* isomerization. The strong dependence of this photoisomerization process on temperature hence confirms that the photoisomerization of FcD allows to populate thermodynamic equilibrium distributions at temperatures where they are not kinetically accessible under thermal activation.

Combining thermal and photochemical stimuli enables switching between two states with different ratios of *E*‐*syn* and *Z*‐*anti*‐FcD. After an initial heating at 100 °C for two days, the thermal equilibrium with an *E* : *Z* ratio of 67 : 33 is reached (Figure 3CII). Subsequent irradiation with 595 nm light at 20 °C for 10 min shifts this *E* : *Z* ratio to 80 : 20 (Figure 3CIII). Another heating period of two days restores the initial *E* : *Z* ratio of 67 : 33 at the thermal equilibrium (Figure 3CIV). These cycles of heating and subsequent irradiation can be repeated multiple times without signs of fatigue or the formation of side or decomposition products (Figure S12), confirming a high degree of reversibility.

### Electrochemistry and Redox Switching

**Figure 4:**
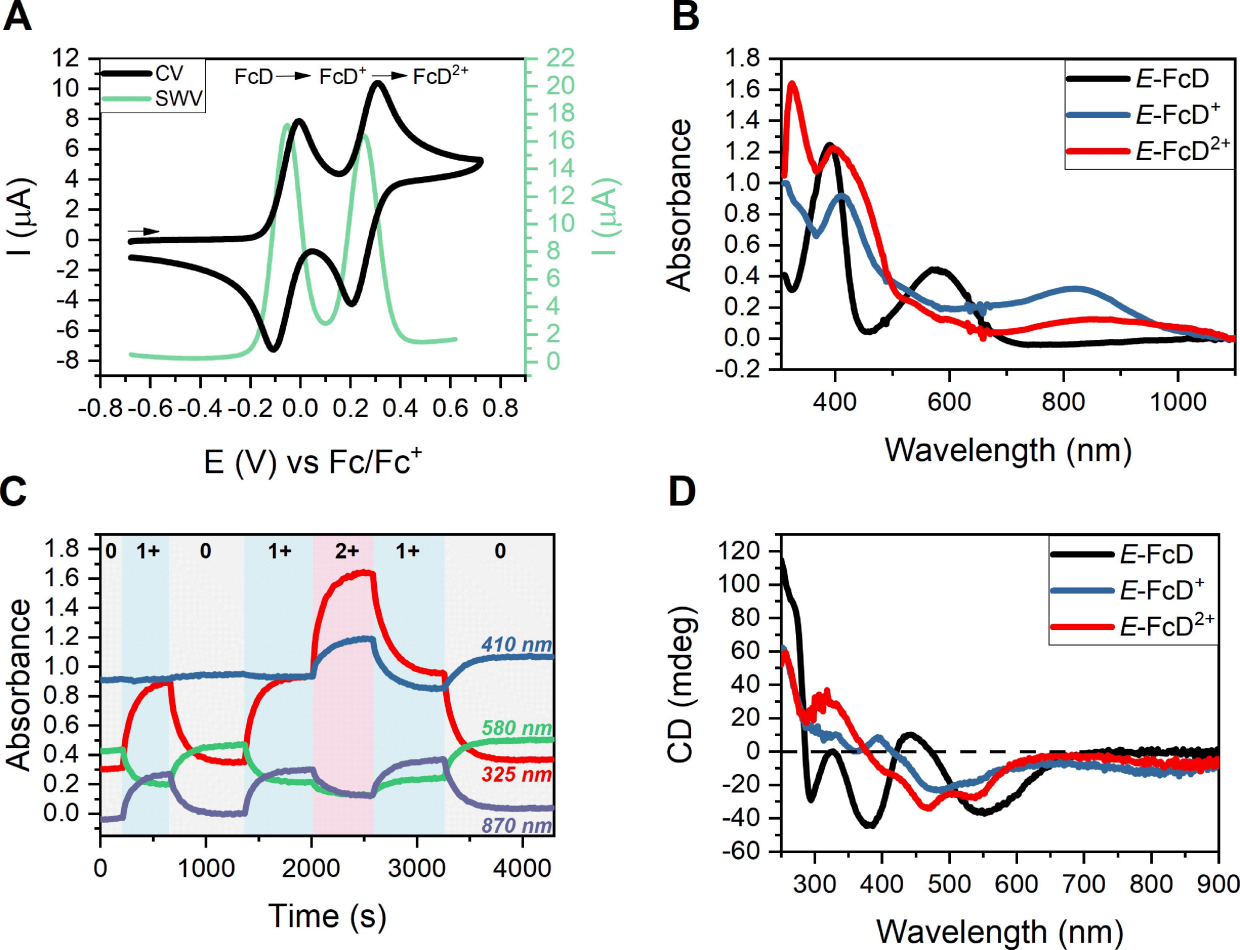
A: CV at 100 mV/s (black) and SWV (green) of 500 μM *E*‐FcD in CH_2_Cl_2_, 100 mM TBAPF_6_. B: UV/Vis spectra of the three oxidation states of *E*‐FcD^0/+/2+^ generated by electrolysis in CH_2_Cl_2_, 200 mM TBAPF_6_. C: The corresponding absorbance time‐traces of spectroelectrochemical cycling between all oxidation states. D: CD spectra of the three oxidation states of 50 μM *E*‐FcD^0/+/2+^ generated by chemical oxidation with magic blue in CH_2_Cl_2_.

The redox properties of *E‐syn*‐FcD were then probed by cyclic voltammetry (CV) and square‐wave voltammetry (SWV) in CH_2_Cl_2_ containing 100 mM TBAPF_6_ as supporting electrolyte. As shown in Figure 4A, the switch displays two reversible one‐electron redox waves corresponding to the sequential oxidation from the neutral *E*‐FcD to the monocationic *E*‐FcD^+^ to the dicationic *E*‐FcD^2+^, with half‐wave potentials of −0.06 and +0.260 V vs. Fc/Fc^+^, respectively. These interconversions occur with a high degree of reversibility and without significant geometric rearrangements as indicated by low peak separations of ~75 mV for both redox couples at a scan rate of 25 mV/s.

Additionally, the excellent linear dependence of the peak currents on the square root of the scan rate confirms that these redox processes are diffusion‐controlled (Figure S22). The large difference in the redox potentials of the first and second oxidation of 320 mV is indicative of significant electronic communication between the two ferrocene motifs and is in good agreement with the conjugated nature of the switch through the central double bond. Importantly, this also allows quantitative generation of the intermediate monocation *E*‐FcD^+^ redox state, which was exploited in spectroelectrochemical studies.

As shown in Figure 4B, bulk potential‐controlled electrolysis enabled facile in situ formation and UV/Vis investigation of *E*‐FcD^+^ and *E*‐FcD^2+^, which both display notable differences in absorbance in comparison to the neutral *E*‐FcD. Specifically, the first oxidation to the monocation is associated with significant changes in the UV/Vis spectrum, such as disappearance of the band at 576 nm in favor of a new, broad, much more red‐shifted absorbance peak at λ_max_=820 nm for *E*‐FcD^+^. The second oxidation to *E*‐FcD^2+^ is associated with additional, albeit smaller, changes in absorbance, including a further red‐shifting and broadening of the lowest‐energy band to λ_max_ ~860 nm which extends to ~1080 nm.

Importantly, all one‐electron interconversions, that is FcD↔FcD^+^ and FcD^+^↔FcD^2+^, proceed via clear isosbestic points in the UV/Vis spectra (Figures S23 and S24), indicative of monomolecular switching with negligible degradation. The latter is also apparent in the low degree of fatigue observed on repeated cycling between the oxidation states, as shown in Figure 4C.

To investigate the influence of oxidation of the switch on its chiral expression, the CD spectra of *E*‐FcD^+^ and *E*‐FcD^2+^ were recorded. In this case, the cationic states were generated by addition of 1 or 2 equivalents of the strong oxidant magic blue (tris(4‐bromophenyl)ammoniumyl hexachloroantimonate). As representatively shown for (*S*,*S*)‐*E*‐FcD in Figure 4D, this is, in analogy to the UV/Vis spectra, associated with significant changes in the intensity and particularly the wavelength of the Cotton bands. For example, both *E‐*FcD^+^ and *E‐*FcD^2+^ display broad CD signals in the near‐IR region (at ~810 and ~870 nm, respectively) which are of identical sign to the most red‐shifted band in the neutral *E‐*FcD. All other bands also change significantly upon oxidation, which, in some cases, is even associated with sign inversion (e.g. at 295, 393 and 440 nm). As expected, the spectra of the enantiomers of *E‐*(*S,S/R,R*)‐FcD^+^ and *E‐*(*S,S/R,R*)‐FcD^2+^ are also mirrored (Figures S25 and S26).

In contrast to other redox‐responsive overcrowded alkenes, such as bisthioxanthylidenes,[^112^, ^113^] where redox processes induce large geometric changes, the changes in chiral expression of *E*‐FcD upon oxidation occur without significant conformational rearrangements. This is, as discussed above, evident from the high degree of electrochemical reversibility (Figure 3A) and is further corroborated by DFT calculations showing very similar geometries for neutral *E*‐FcD and oxidized *E*‐FcD^+^ (see Figures S1–S9). Specifically, the calculated lowest energy conformer of (*R*,*R*)‐*E*‐FcD^+^ retains the (*M*,*M*)‐helicity observed in the neutral state. A distribution of (*M*,*M*)‐(*R*,*R*)‐*E*‐FcD^+^ : (*P*,*P*)‐(*R*,*R*)‐*E*‐FcD^+^ in a ratio of 98 : 2 was calculated at 20 °C (see Table S2), identical to the one obtained for the corresponding neutral states. The activation energy for this helicity inversion process is also very similar to the one calculated for the neutral state with a Gibbs free energy of activation ΔG^≠^_THI‐*E‐syn‐*FcD_^+^=40.1 kJ/mol at 25 °C.

These observations highlight that *E*‐*syn*‐FcD is a potent redox‐driven chiroptical switch with three stable and quantitatively accessible redox states that all possess different chiroptical properties.

### *E*‐*syn‐*FcD as Redox‐Switchable LC Dopant

**Figure 5:**
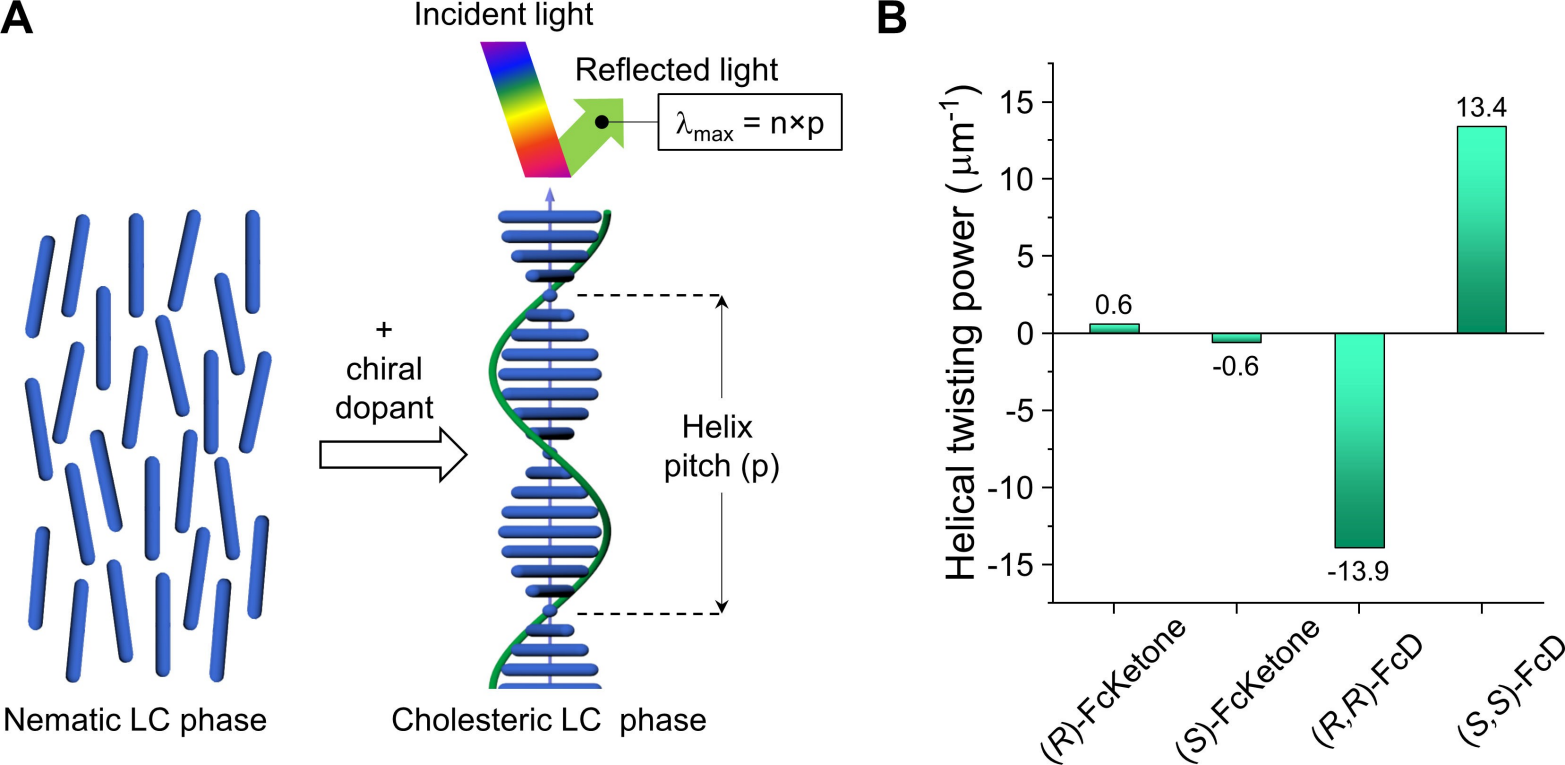
A: Schematic representation of the cholesteric LC phase induction by doping a nematic phase with chiral molecules (dopants). The periodicity of the supramolecular helical architecture (p) acts as a Bragg diffraction grating, leading to light reflection of a specific color (λ_max_) and circular polarization which is defined by the handedness of the helix. B: Helical twisting power values of the ferrocene dimers and their corresponding planar chiral ketones. The positive values correspond to a right‐handed cholesteric helix, while the negative values correspond to a left‐handed helix. The values were calculated based on wt% concentration.

One of the simplest ways to obtain a cholesteric LC phase is the doping of nematic LCs with small amounts of chiral additives (or dopants) as shown in Figure 5A. The supramolecular helical organization of the cholesteric phase has a distinctive optical property, the selective reflection of light, where normally incident light of a specific wavelength (λ_max_) and circular polarization is reflected by the helical structure and the color is defined by the helix pitch (p): λ_max_=np, where *n* is the average refractive index of the material. The strength with which the chiral dopant twists LCs is characterized by the HTP, which can be calculated by the equation: HTP=p^−1^/c, where *c* is the concentration of the chiral dopant. The HTP value encompasses both the chiral shape of the dopant and its interactions with the surrounding LC molecules, which opens a wide range of possibilities for manipulating the optical properties of the cholesteric material.

As mentioned before, in order to manipulate the optical properties of cholesteric LCs by a redox process, Aida and co‐workers reported the use of a ferrocene moiety coupled with an axially chiral BINOL fragment via an aliphatic spacer. For the initial (reduced) state of the dopant, the HTP was measured to be 116 μm^−1^, whereas its oxidation led to a decrease in HTP to 101 μm^−1^ (or by approx. 13 %). Consequently, the authors achieved a reversible change in the selective light reflection wavelength of the cholesteric material of 18 nm by electrochemical redox‐switching in an electro‐optic cell.^[91]^ In contrast to this design where the redox active unit is separated from the chiral motif and therefore has limited influence, a distinctive feature of FcD is that it is intrinsically chiral with two planar chiral ferrocene units forming dynamic helical chirality, which may be beneficial for its ability to tune the properties of cholesteric LC materials.

To study the twisting ability of both the *E*‐*syn*‐FcD dimers and their ketone precursors, the Grandjean‐Cano wedge method was used (see details in Supporting Information).[^114^, ^115^, ^116^] Doping of the nematic LC E7 with 2 wt % of enantiopure ketones (*R*)‐FcKetone and (*S*)‐FcKetone, respectively, possessing only the planar chirality of ferrocene, leads to twisting of the cholesteric helical structure (p~81–86 μm) and is characterized by similar low values of HTP (0.6 μm^−1^) which are opposite in sign (Figure 5B, Figure S29).

Both dimers showed significantly higher HTP values exceeding 13 μm^−1^. Additionally, the sign of the helix induced by the dimer is opposite to the sign of the helix formed by the corresponding ketone (Figure 5B, Figure S28). Thus, doping with (*R*,*R*)‐FcD induces the left‐handed helix and (*R*)‐FcKetone the right‐handed one. This indicates that the axial chirality of the dimer is much more efficiently transferred to LC molecules compared with the planar chirality of the ferrocene moieties, which is in line with literature precedents where axial or helical chirality outcompetes any other type of chirality present in the structure of the studied dopants.[^117^, ^118^]

**Figure 6:**
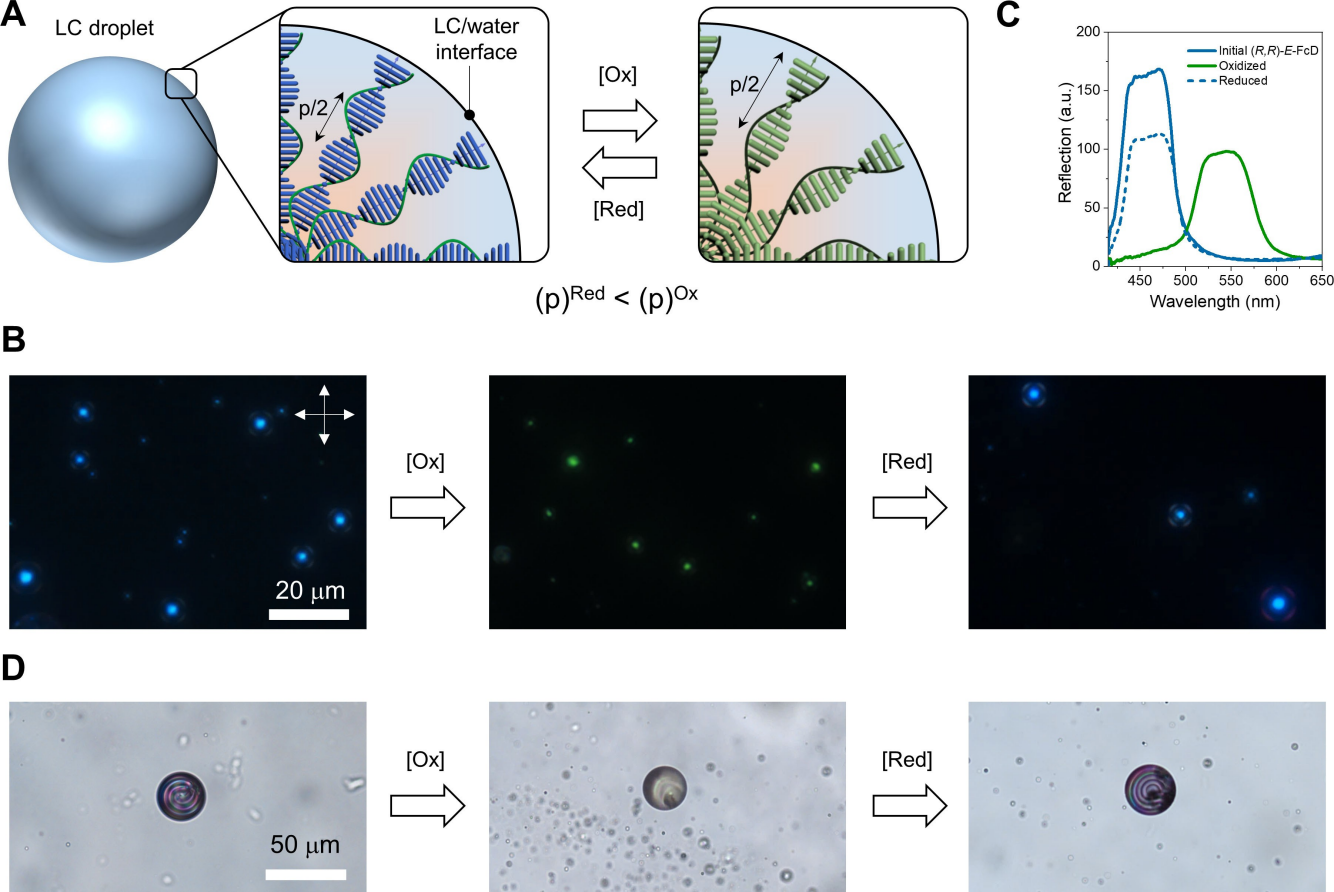
A: Schematic representation of the LC droplet with a radial helical configuration in water, subjected to a reversible oxidation–reduction reaction at the LC‐water interface. B: Polarized optical images (acquired in reflection mode) of cholesteric droplets ((*R*,*R*)‐*E*‐FcD/(*R*)‐BB/E7: 4/5/91) in a water PVA solution. The droplets were oxidized and reduced by incubation in aqueous iron (III) perchlorate or ascorbic acid solutions, respectively. The white arrows correspond to the crossed polarizers. C: Reflection spectra of the same redox‐active cholesteric mixture after a full cycle of oxidation/reduction. D: Bright field optical images of the LC droplets made of a cholesteric mixture with a long helix pitch ((*R*,*R*)‐*E*‐FcD, 2 wt % in E7) before oxidation, after oxidation, and after subsequent reduction.

In contrast to electrochemical oxidation/reduction performed directly in electro‐optical cells, which is a rather elaborate procedure and requires the addition of a supporting electrolyte that negatively affects the LC phase itself and the alignment of molecules at interfaces, a chemical in situ oxidation/reduction procedure was used in this study. For this purpose, LC droplets^[119]^ in water were prepared and stabilized with poly(vinylalcohol) (PVA), which also promotes the planar orientation of the LC molecules at the interface with water so that the axes of the supramolecular helices have a radial orientation. It is known that cholesteric LCs confined in spherical geometries demonstrate different optical fingerprints^[120]^ depending on the helix pitch. To demonstrate how in situ redox reactions affect the cholesteric helices and eventually optical properties of the LC droplets two mixtures characterized by either a short (few hundreds nm) or a long (several μm) helix pitch were fabricated. In spite of the overall moderate HTP values of the chiral dimers and their rather good solubility in LCs (up to 5 wt %) an additional “passive”, i.e. non‐responsive, chiral dopant in order to reach a nanometer scale pitch which corresponds to selective reflection of visible light had to be used. The mixture of 4 wt % (*R*,*R*)‐FcD in E7 was doped with additional 5 wt % (*R*)‐BB (bridged‐BINOL dopant which induces the same helical twist as (*R*,*R*)‐FcD, see details in Supporting Information), to create a permanent offset pitch. The droplets made of this mixture selectively reflect blue light that can be observed using polarizing optical microscopy (Figure 6A, B). Only the central area of the isolated droplet is colored under normal incident light due to omnidirectional Bragg reflection.^[121]^ When the droplets were exposed to an aqueous solution of Fe(ClO_4_)_3_ for 5 min, oxidation of (*R*,*R*)‐FcD at the droplet interface occurred and due to their small size (<10 μm), the droplets quickly changed their color to green (Figure 6B). Such a strong color change suggests that the HTP of (*R*,*R*)‐FcD^+^ tends towards zero and only the passive (*R*)‐BB dopant now determines the pitch of the cholesteric helix.

Although the axial chirality remains preserved upon oxidation of (*R*,*R*)‐FcD, as confirmed by CD data (Figure S26), the HTP dramatically drops. We propose that in the neutral state of FcD, its chirality is mainly transduced to the LC through π‐interactions. However, upon oxidation, additional strong ion‐dipole interactions, between the dopant and the terminal CN‐group of the LC, might dominate over the π‐interactions, which could prevent the specific orientation of LC molecules around the chiral dopant, necessary for efficient chirality transfer. This mechanism is consistent with the behavior of previously reported chiral ferrocenes.^[91]^ The virtually zero HTP values of FcD^+^ were also confirmed by independent measurements (see details in Supporting Information, Figure S30).

Droplets containing (*R*,*R*)‐FcD^+^ can be easily reduced back to (*R*,*R*)‐FcD by placing them in an aqueous solution of ascorbic acid, which results in the twisting of the cholesteric helix and complete recovery of the blue color of the droplets (Figure 6B). A study of this redox‐switching process of the cholesteric mixture was also performed on a supported LC layer by measuring its light reflectance directly under a microscope (see details in Supporting Information, Figure S33). The reflection spectra of the mixture after a complete oxidation/reduction cycle are shown in Figure 6C and confirm the reversible redox behavior of (*R*,*R*)‐FcD. It can also be seen that the shift of the position of the reflection band is as much as 84 nm, which is significantly higher than the values achieved so far.[^91^, ^92^] Importantly, almost full recovery of the initial reflectance wavelength was observed upon reduction, highlighting the reversibility of the redox switching process.

It is worth emphasizing that the color change of the droplets is solely tuned by the oxidation/reduction reaction of the chiral ferrocene and no other components of the LC mixture are involved in this process, which was demonstrated by a control mixture lacking (*R*,*R*)‐FcD and containing only (*R*)‐BB (Figure S31).

To demonstrate how the redox process influences the optical properties of cholesteric droplets with a long helix pitch, a mixture containing only 2 wt % of (*R*)‐FcD dissolved in E7 was prepared which is characterized by a helix pitch of about 3.6 μm. In the initial state, the chiral (*R*,*R*)‐FcD‐doped LC droplet has a typical spiral‐like optical texture^[122]^ which completely disappears upon oxidation (25 mM Fe(ClO_4_)_3_, 30 min). This indicates a loss of chirality (i.e. the cholesteric helix is fully unwound, the HTP is reaching zero value) which can be clearly seen in Figure 6D. Moreover, the purple color of the droplet turns brown confirming the formation of (*R*,*R*)‐FcD^+^. The spiral‐like optical texture as well as the color of the droplet was fully restored upon subsequent reduction (50 mM ascorbic acid aqueous solution, 1 h). The control mixture containing no FcD did not demonstrate any textural changes under oxidative conditions (Figure S32) confirming that FcD is responsible for the redox modulation of the cholesteric supramolecular structure.

Preliminary tests further demonstrated that FcD responds to light when embedded into LCs. However, the range in which the HTP can be switched in this manner is rather small, as the range of photochemical or thermal equilibria between *E*‐*syn*‐FcD and *Z*‐*anti*‐FcD is limited (more details as well as an estimation of the HTP values of the *Z*‐*anti* form of FcD are presented in the Supporting Information, Figure S34).

Thus, the new design of the chiral redox‐active dopants developed herein enables reversible modulation of the HTP values within an unprecedentedly wide range (from −13.9 μm^−1^ to −1.2 μm^−1^, corresponding to a >90 % change). Such compounds have potential applications as multi‐stimuli‐responsive chiral additives for LC‐based tunable optical materials.

## Conclusion

The construction of stimuli‐responsive switches with well‐defined geometry and chirality modulations remains an important but difficult challenge. Herein, we demonstrated that FcD, a dimer of a simple planar chiral ferrocene building block, is a versatile molecular switch that is easily synthetically accessible in excellent yield and enantiopurity. Remarkably, during the final dimerization step, the relative orientation of the ferrocene motifs (*syn*), the double bond geometry (*E*) and the helicity of the switch are fixed and only a single switch isomer is formed from each enantiomeric precursor in a fully stereocontrolled manner. Due to the two ferrocene motifs, the switch not only displays the ability to interconvert between three oxidation states with high reversibility but is also deeply purple colored with absorbance in the near IR region. As a result, light of up to 780 nm, or heat, can also be used to (optically) actuate the central overcrowded alkene bond, inducing *E/Z* isomerization from *E*‐*syn*‐FcD to *Z*‐*anti*‐FcD, albeit with only relatively low conversions. In contrast, quantitative oxidation to either the mono‐ or dicationic state is possible both chemically and electrochemically and is associated with significant red‐shifts of the absorbance as well as changes in its chiral properties as shown by CD spectroscopy. This process is not only highly reversible, but also significantly alters the HTP of the switch when used as a chiral dopant in a nematic LC. Specifically, the initial HTP of 13 μm^−1^ of *E*‐*syn*‐FcD is reduced to near zero upon oxidation to *E*‐*syn*‐FcD^+^, which is associated with significant changes in the reflection color of the LC. This was demonstrated by chemical redox switching of FcD‐doped LC droplets suspended in water, whose initial blue color changes to green upon oxidation of the dopant, corresponding to an 84 nm shift in reflection color, the largest value attained thus far using a redox‐responsive dopant in a LC material. Due to the simple synthetic methodology, significant amounts of enantiopure dopant can be prepared without the need for laborious enantioseparation, thereby opening up many opportunities for further applications of such multi‐stimuli‐responsive (redox, heat, and light) intrinsically chiral modifiers, well beyond LCs but also in numerous other applications where (redox) chiroptical switching is required.

## Conflict of Interests

The authors declare no conflict of interest.

1

## Supporting information

As a service to our authors and readers, this journal provides supporting information supplied by the authors. Such materials are peer reviewed and may be re‐organized for online delivery, but are not copy‐edited or typeset. Technical support issues arising from supporting information (other than missing files) should be addressed to the authors.

Supporting Information

Supporting Information

## Data Availability

The data that support the findings of this study are available in the supplementary material of this article.

## References

[1] B. L. Feringa , R. A. Van Delden , N. Koumura , E. M. Geertsema , Chem. Rev. 2000, 100, 1789–1816. 11777421 10.1021/cr9900228

[2] W. R. Browne , B. L. Feringa , in Molecular Switches, Wiley-VCH, Weinheim, Germany, 2011.

[3] D. Bléger , S. Hecht , Angew. Chem. Int. Ed. 2015, 54, 11338–11349. 10.1002/anie.201500628 26096635

[4] Z. L. Pianowski , in Molecular Photoswitches: Chemistry, Properties, and Applications, Wiley-VCH, Germany, 2022.

[5] B. L. Feringa , Angew. Chem. Int. Ed. 2017, 56, 11060–11078. 10.1002/anie.201702979 28851050

[6] L. A. Huber , K. Hoffmann , S. Thumser , N. Böcher , P. Mayer , H. Dube , Angew. Chem. 2017, 129, 14728–14731. 10.1002/anie.201708178 28892243

[7] S. Kassem , T. Van Leeuwen , A. S. Lubbe , M. R. Wilson , B. L. Feringa , D. A. Leigh , Chem. Soc. Rev. 2017, 46, 2592–2621. 28426052 10.1039/c7cs00245a

[8] R. D. Astumian , Chem. Sci. 2017, 8, 840–845. 28572896 10.1039/c6sc04806d PMC5452271

[9] D. Roke , S. J. Wezenberg , B. L. Feringa , Proc. Nat. Acad. Sci. 2018, 115, 9423–9431. 29712825 10.1073/pnas.1712784115 PMC6156651

[10] J. C. M. Kistemaker , A. S. Lubbe , B. L. Feringa , Mater. Chem. Front. 2021, 5, 2900–2906.

[11] D. R. S. Pooler , A. S. Lubbe , S. Crespi , B. L. Feringa , Chem. Sci. 2021, 12, 14964–14986. 34909140 10.1039/d1sc04781g PMC8612399

[12] V. Balzani , A. Credi , M. Venturi , in Molecular Devices and Machines: Concepts and Perspectives for the Nanoworld, Wiley-VCH, Weinheim, Germany, 2008.

[13] V. Balzani , A. Credi , F. M. Raymo , J. F. Stoddart , Angew. Chem. 2000, 39, 3348–3391. 11091368 10.1002/1521-3773(20001002)39:19<3348::aid-anie3348>3.0.co;2-x

[14] B. Champin , P. Mobian , J.-P. Sauvage , Chem. Soc. Rev. 2007, 36, 358–366. 17264936 10.1039/b604484k

[15] J. F. Stoddart , Angew. Chem. Int. Ed. 2017, 56, 11094–11125. 10.1002/anie.201703216 28815900

[16] S. Erbas-Cakmak , D. A. Leigh , C. T. McTernan , A. L. Nussbaumer , Chem. Rev. 2015, 115, 10081–10206. 26346838 10.1021/acs.chemrev.5b00146 PMC4585175

[17] K. Kinbara , T. Aida , Chem. Rev. 2005, 105, 1377–1400. 15826015 10.1021/cr030071r

[18] R. Herges , Chem. Sci. 2020, 11, 9048–9055. 34123157 10.1039/d0sc03094e PMC8163427

[19] T. Muraoka , K. Kinbara , T. Aida , Nature 2006, 440, 512–515. 16554815 10.1038/nature04635

[20] I. Aprahamian , ACS Cent. Sci. 2020, 6, 347–358. 32232135 10.1021/acscentsci.0c00064 PMC7099591

[21] A. Livoreil , C. O. Dietrich-Buchecker , J.-P. Sauvage , J. Am. Chem. Soc. 1994, 116, 9399–9400. 27715033 10.1021/ja00099a095

[22] C. J. Bruns , J. F. Stoddart , Acc. Chem. Res. 2014, 47, 2186–2199. 24877992 10.1021/ar500138u

[23] J. Chen , F. K.-C. Leung , M. C. A. Stuart , T. Kajitani , T. Fukushima , E. Van Der Giessen , B. L. Feringa , Nat. Chem. 2018, 10, 132–138. 29359757 10.1038/nchem.2887

[24] F. Lancia , A. Ryabchun , A.-D. Nguindjel , S. Kwangmettatam , N. Katsonis , Nat. Commun. 2019, 10, 4819. 31645565 10.1038/s41467-019-12786-2 PMC6811622

[25] L. Feng , Y. Qiu , Q.-H. Guo , Z. Chen , J. S. W. Seale , K. He , H. Wu , Y. Feng , O. K. Farha , R. D. Astumian , J. F. Stoddart , Science 2021, 374, 1215–1221. 34672694 10.1126/science.abk1391

[26] Y. Feng , M. Ovalle , J. S. W. Seale , C. K. Lee , D. J. Kim , R. D. Astumian , J. F. Stoddart , J. Am. Chem. Soc. 2021, 143, 5569–5591. 33830744 10.1021/jacs.0c13388

[27] S. Amano , S. D. P. Fielden , D. A. Leigh , Nature 2021, 594, 529–534. 34163057 10.1038/s41586-021-03575-3

[28] S. Corra , M. T. Bakić , J. Groppi , M. Baroncini , S. Silvi , E. Penocchio , M. Esposito , A. Credi , Nat. Nanotechnol. 2022, 17, 746–751. 35760895 10.1038/s41565-022-01151-y

[29] A. Koçer , M. Walko , W. Meijberg , B. L. Feringa , Science 2005, 309, 755–758. 16051792 10.1126/science.1114760

[30] Y. Qutbuddin , A. Guinart , S. Gavrilović , K. Al Nahas , B. L. Feringa , P. Schwille , Adv. Mater. 2024, 36, 2311176. 10.1002/adma.202311176 38215457

[31] J. Chen , S. J. Wezenberg , B. L. Feringa , Chem. Commun. 2016, 52, 6765–6768. 10.1039/c6cc02382g 27068214

[32] S. Kassem , A. T. L. Lee , D. A. Leigh , A. Markevicius , J. Solà , Nat. Chem. 2016, 8, 138–143. 26791896 10.1038/nchem.2410

[33] J.-P. Collin , C. Dietrich-Buchecker , P. Gaviña , M. C. Jimenez-Molero , J.-P. Sauvage , Acc. Chem. Res. 2001, 34, 477–487. 11412084 10.1021/ar0001766

[34] S. Silvi , M. Venturi , A. Credi , J. Mater. Chem. 2009, 19, 2279.

[35] A. M. Schoevaars , W. Kruizinga , R. W. J. Zijlstra , N. Veldman , A. L. Spek , B. L. Feringa , J. Org. Chem. 1997, 62, 4943–4948.

[36] K. K. Kartha , A. Takai , Z. Futera , J. Labuta , M. Takeuchi , Angew. Chem. Int. Ed. 2021, 60, 16466–16471. 10.1002/anie.202102719 33905168

[37] A. Gerwien , F. Gnannt , P. Mayer , H. Dube , Nat. Chem. 2022, 14, 670–676. 35437331 10.1038/s41557-022-00917-0

[38] M. Querol , H. Stoekli-Evans , P. Belser , Org. Lett. 2002, 4, 1067–1070. 11922784 10.1021/ol017130c

[39] M. L. C. M. Oosterling , A. M. Schoevaars , H. J. Haitjema , B. L. Feringa , Isr. J. Chem. 1996, 36, 341–348.

[40] N. Koumura , R. W. J. Zijlstra , R. A. Van Delden , N. Harada , B. L. Feringa , Nature 1999, 401, 152–155. 10490022 10.1038/43646

[41] C. Kammerer , G. Erbland , Y. Gisbert , T. Nishino , K. Yasuhara , G. Rapenne , Chem. Lett. 2019, 48, 299–308.

[42] V. García-López , D. Liu , J. M. Tour , Chem. Rev. 2020, 120, 79–124. 31849216 10.1021/acs.chemrev.9b00221

[43] V. García-López , F. Chen , L. G. Nilewski , G. Duret , A. Aliyan , A. B. Kolomeisky , J. T. Robinson , G. Wang , R. Pal , J. M. Tour , Nature 2017, 548, 567–572. 28858304 10.1038/nature23657

[44] A. Guinart , M. Korpidou , D. Doellerer , G. Pacella , M. C. A. Stuart , I. A. Dinu , G. Portale , C. Palivan , B. L. Feringa , Proc. Nat. Acad. Sci. 2023, 120, e2301279120. 37364098 10.1073/pnas.2301279120 PMC10319042

[45] W. Danowski , T. Van Leeuwen , S. Abdolahzadeh , D. Roke , W. R. Browne , S. J. Wezenberg , B. L. Feringa , Nat. Nanotechnol. 2019, 14, 488–494. 30886378 10.1038/s41565-019-0401-6

[46] S. Krause , B. L. Feringa , Nat. Chem. Rev. 2020, 4, 550–562.

[47] C. Stähler , L. Grunenberg , M. W. Terban , W. R. Browne , D. Doellerer , M. Kathan , M. Etter , B. V. Lotsch , B. L. Feringa , S. Krause , Chem. Sci. 2022, 13, 8253–8264. 35919721 10.1039/d2sc02282f PMC9297439

[48] J. Sheng , J. Perego , W. Danowski , S. Bracco , S. Chen , X. Zhu , C. X. Bezuidenhout , S. Krause , W. R. Browne , P. Sozzani , A. Comotti , B. L. Feringa , Chem 2023, 9, 2701–2716.

[49] R. A. Van Delden , M. K. J. Ter Wiel , M. M. Pollard , J. Vicario , N. Koumura , B. L. Feringa , Nature 2005, 437, 1337–1340. 16251960 10.1038/nature04127

[50] G. London , G. T. Carroll , T. Fernández Landaluce , M. M. Pollard , P. Rudolf , B. L. Feringa , Chem. Commun. 2009, 1712. 10.1039/b821755f 19294271

[51] R. A. Van Delden , N. Koumura , N. Harada , B. L. Feringa , Proc. Nat. Acad. Sci. 2002, 99, 4945–4949. 11929969 10.1073/pnas.062660699 PMC122700

[52] J. Hou , G. Long , W. Zhao , G. Zhou , D. Liu , D. J. Broer , B. L. Feringa , J. Chen , J. Am. Chem. Soc. 2022, 144, 6851–6860. 35380815 10.1021/jacs.2c01060 PMC9026258

[53] J. Li , S. Xie , J. Meng , Y. Liu , Q. Zhan , Y. Zhang , L. Shui , G. Zhou , B. L. Feringa , J. Chen , CCS Chem. 2023, 1–12.

[54] G. Long , Y. Deng , W. Zhao , G. Zhou , D. J. Broer , B. L. Feringa , J. Chen , J. Am. Chem. Soc. 2024, 146, 13894–13902. 38728606 10.1021/jacs.4c01642 PMC11117400

[55] P. Štacko , J. C. M. Kistemaker , T. Van Leeuwen , M.-C. Chang , E. Otten , B. L. Feringa , Science 2017, 356, 964–968. 28572394 10.1126/science.aam8808

[56] C. L. F. Van Beek , B. L. Feringa , J. Am. Chem. Soc. 2024, 146, 5634–5642. 38350104 10.1021/jacs.3c14430 PMC10910502

[57] J. Wang , B. L. Feringa , Science 2011, 331, 1429–1432. 21310964 10.1126/science.1199844

[58] S. F. Pizzolato , P. Štacko , J. C. M. Kistemaker , T. Van Leeuwen , E. Otten , B. L. Feringa , J. Am. Chem. Soc. 2018, 140, 17278–17289. 30458108 10.1021/jacs.8b10816 PMC6326533

[59] Q. Li , G. Fuks , E. Moulin , M. Maaloum , M. Rawiso , I. Kulic , J. T. Foy , N. Giuseppone , Nat. Nanotechnol. 2015, 10, 161–165. 25599191 10.1038/nnano.2014.315

[60] E. Moulin , L. Faour , C. C. Carmona-Vargas , N. Giuseppone , Adv. Mater. 2020, 32, 1906036. 10.1002/adma.201906036 31833132

[61] F. Xu , L. Pfeifer , S. Crespi , F. K.-C. Leung , M. C. A. Stuart , S. J. Wezenberg , B. L. Feringa , J. Am. Chem. Soc. 2021, 143, 5990–5997. 33830767 10.1021/jacs.1c01802 PMC8154511

[62] B. L. Feringa , W. F. Jager , B. De Lange , Tetrahedron 1993, 49, 8267–8310.

[63] I. Agranat , H. Caner , J. Caldwell , Nat. Rev. Drug Discovery 2002, 1, 753–768. 12360254 10.1038/nrd915

[64] Y.-N. Qin , C. Zhang , Q. Li , G.-Y. Du , Synthesis 2021, 53, 4588–4598.

[65] M. K. J. Ter Wiel , R. A. van Delden , A. Meetsma , B. L. Feringa , J. Am. Chem. Soc. 2003, 125, 15076–15086. 14653742 10.1021/ja036782o

[66] M. Guentner , M. Schildhauer , S. Thumser , P. Mayer , D. Stephenson , P. J. Mayer , H. Dube , Nat. Commun. 2015, 6, 8406. 26411883 10.1038/ncomms9406 PMC4598625

[67] M. K. J. Ter Wiel , N. Koumura , R. A. Van Delden , A. Meetsma , N. Harada , B. L. Feringa , Chirality 2000, 12, 734–741. 11054832 10.1002/1520-636X(2000)12:10<734::AID-CHIR6>3.0.CO;2-Q

[68] L. F. Tietze , A. Düfert , F. Lotz , L. Sölter , K. Oum , T. Lenzer , T. Beck , R. Herbst-Irmer , J. Am. Chem. Soc. 2009, 131, 17879–17884. 19911798 10.1021/ja906260x

[69] H. Liu , M. El-Salfiti , M. Lautens , Angew. Chem. Int. Ed. 2012, 51, 9846–9850. 10.1002/anie.201204226 22926928

[70] T. C. Pijper , D. Pijper , M. M. Pollard , F. Dumur , S. G. Davey , A. Meetsma , B. L. Feringa , J. Org. Chem. 2010, 75, 825–838. 20055375 10.1021/jo902348u

[71] T. M. Neubauer , T. Van Leeuwen , D. Zhao , A. S. Lubbe , J. C. M. Kistemaker , B. L. Feringa , Org. Lett. 2014, 16, 4220–4223. 25079823 10.1021/ol501925f PMC5055213

[72] T. Van Leeuwen , W. Danowski , E. Otten , S. J. Wezenberg , B. L. Feringa , J. Org. Chem. 2017, 82, 5027–5033. 28459576 10.1021/acs.joc.7b00852 PMC5442604

[73] Y. Gisbert , M. Fellert , C. N. Stindt , A. Gerstner , B. L. Feringa , J. Am. Chem. Soc. 2024, 146, 12609–12619. 38656891 10.1021/jacs.4c01628 PMC11082891

[74] Q. Li , J. T. Foy , J.-R. Colard-Itté , A. Goujon , D. Dattler , G. Fuks , E. Moulin , N. Giuseppone , Tetrahedron 2017, 73, 4874–4882. 10.1038/nnano.2017.28 28319615

[75] R. López , C. Palomo , Angew. Chem. Int. Ed. 2022, 61, e202113504. 10.1002/anie.202113504 PMC9304569 34717037

[76] D. Coates , Liq. Cryst. 2015, 1–13.

[77] M. Mitov , Soft Matter 2017, 13, 4176–4209. 28589190 10.1039/c7sm00384f

[78] A. Ryabchun , A. Bobrovsky , Adv. Opt. Mater. 2018, 6, 1800335.

[79] H. K. Bisoyi , Q. Li , Chem. Rev. 2022, 122, 4887–4926. 34941251 10.1021/acs.chemrev.1c00761

[80] B. L. Feringa , N. P. M. Huck , H. A. Van Doren , J. Am. Chem. Soc. 1995, 117, 9929–9930.

[81] R. Eelkema , M. M. Pollard , N. Katsonis , J. Vicario , D. J. Broer , B. L. Feringa , J. Am. Chem. Soc. 2006, 128, 14397–14407. 17076514 10.1021/ja065334o

[82] Q. Li , L. Green , N. Venkataraman , I. Shiyanovskaya , A. Khan , A. Urbas , J. W. Doane , J. Am. Chem. Soc. 2007, 129, 12908–12909. 17927184 10.1021/ja0747573

[83] D. Pijper , M. G. M. Jongejan , A. Meetsma , B. L. Feringa , J. Am. Chem. Soc. 2008, 130, 4541–4552. 18335940 10.1021/ja711283c

[84] T. J. White , R. L. Bricker , L. V. Natarajan , N. V. Tabiryan , L. Green , Q. Li , T. J. Bunning , Adv. Funct. Mater. 2009, 19, 3484–3488.

[85] Z. Zheng , H. Hu , Z. Zhang , B. Liu , M. Li , D.-H. Qu , H. Tian , W.-H. Zhu , B. L. Feringa , Nat. Photonics 2022, 16, 226–234.

[86] J. Hou , J. Wang , A. Ryabchun , B. L. Feringa , Adv. Funct. Mater. 2024, 34, 2312831.

[87] R. Eelkema , M. M. Pollard , J. Vicario , N. Katsonis , B. S. Ramon , C. W. M. Bastiaansen , D. J. Broer , B. L. Feringa , Nature 2006, 440, 163–163. 16525460 10.1038/440163a

[88] Y. Wang , Q. Li , Adv. Mater. 2012, 24, 1926–1945. 22411073 10.1002/adma.201200241

[89] T. J. White , S. A. Cazzell , A. S. Freer , D. Yang , L. Sukhomlinova , L. Su , T. Kosa , B. Taheri , T. J. Bunning , Adv. Mater. 2011, 23, 1389–1392. 21400602 10.1002/adma.201003577

[90] A. Ryabchun , F. Lancia , J. Chen , D. Morozov , B. L. Feringa , N. Katsonis , Adv. Mater. 2020, 32, 2004420. 10.1002/adma.202004420 33073425

[91] S. Tokunaga , Y. Itoh , H. Tanaka , F. Araoka , T. Aida , J. Am. Chem. Soc. 2018, 140, 10946–10949. 30070108 10.1021/jacs.8b06323

[92] Y. Zhang , W. He , Y. Cui , L. Zhang , Y. Li , Z. Yang , D. Wang , H. Cao , Liq. Cryst. 2022, 49, 1901–1911.

[93] Y. Itoh , D. Morishita , Polym. J. 2023, 55, 1035–1048.

[94] P. U. Biedermann , A. Levy , J. J. Stezowski , I. Agranat , Chirality 1995, 7, 199–205.

[95] P. Denifl , A. Hradsky , B. Bildstein , K. Wurst , J. Organomet. Chem. 1996, 523, 79–91.

[96] D.-W. Gao , Q. Yin , Q. Gu , S.-L. You , J. Am. Chem. Soc. 2014, 136, 4841–4844. 24625115 10.1021/ja500444v

[97] R. Deng , Y. Huang , X. Ma , G. Li , R. Zhu , B. Wang , Y.-B. Kang , Z. Gu , J. Am. Chem. Soc. 2014, 136, 4472–4475. 24617772 10.1021/ja500699x

[98] G. Mlostoń , P. Pipiak , R. Hamera-Fałdyga , H. Heimgartner , Beilstein J. Org. Chem. 2017, 13, 1900–1906. 29062409 10.3762/bjoc.13.185 PMC5629374

[99] G. Mlostoń , R. Hamera-Fałdyga , H. Heimgartner , J. Sulfur Chem. 2018, 39, 267–278. 10.1039/c8ob01022f 29850725

[100] B. P. Corbet , M. B. S. Wonink , B. L. Feringa , Chem. Commun. 2021, 57, 7665–7668. 10.1039/d1cc03098a PMC8330637 34254090

[101] S. Grimme , A. Hansen , S. Ehlert , J.-M. Mewes , J. Chem. Phys. 2021, 154, 064103. 33588555 10.1063/5.0040021

[102] V. Barone , M. Cossi , J. Phys. Chem. A 1998, 102, 1995–2001.

[103] K. Fukunaga , Synthesis 1975, 1975, 442–443.

[104] X. Xia , H. Yu , L. Wang , Z. ul-Abdin , RSC Adv. 2016, 6, 105296–105316.

[105] S. Liu , J. Wang , J. Li , M. Chen , S. Yang , Inorg. Chim. Acta 2009, 362, 4174–4178.

[106] J. R. Horsley , X. Wang , J. Yu , A. D. Abell , Electrochim. Acta 2021, 381, 138232.

[107] F. Bejarano , D. Gutiérrez , J. Catalán-Toledo , D. Roca-Sanjuán , J. Gierschner , J. Veciana , M. Mas-Torrent , C. Rovira , N. Crivillers , Phys. Chem. Chem. Phys. 2022, 24, 6185–6192. 35229090 10.1039/d1cp05012e

[108] J. Park , Y. Choi , S. S. Lee , J. H. Jung , Org. Lett. 2019, 21, 1232–1236. 30730150 10.1021/acs.orglett.9b00277

[109] H. Zheng , L. Fu , R. Wang , J. Jiao , Y. Song , C. Shi , Y. Chen , J. Jiang , C. Lin , J. Ma , L. Wang , Nat. Commun. 2023, 14, 590. 36737437 10.1038/s41467-023-36131-w PMC9898256

[110] K. Takaishi , A. Muranaka , M. Kawamoto , M. Uchiyama , J. Org. Chem. 2011, 76, 7623–7628. 21812476 10.1021/jo201578z

[111] R. Thomas , Y. Yoshida , T. Akasaka , N. Tamaoki , Chem. Eur. J. 2012, 18, 12337–12348. 22907600 10.1002/chem.201200836

[112] W. R. Browne , M. M. Pollard , B. De Lange , A. Meetsma , B. L. Feringa , J. Am. Chem. Soc. 2006, 128, 12412–12413. 16984180 10.1021/ja064423y

[113] M. B. S. Wonink , B. P. Corbet , A. A. Kulago , G. B. Boursalian , B. De Bruin , E. Otten , W. R. Browne , B. L. Feringa , J. Am. Chem. Soc. 2021, 143, 18020–18028. 34695359 10.1021/jacs.1c05938 PMC8569810

[114] F. Grandjean , C. R. Seances Acad. Sci. Paris 1921, 172, 71.

[115] R. Cano , Bull. Minéralogie 1968, 91, 20–27.

[116] P. R. Gerber , Z. Für Naturforschung A 1980, 35, 619–622.

[117] R. Eelkema , B. L. Feringa , Org. Biomol. Chem. 2006, 4, 3729. 17024276 10.1039/b608749c

[118] N. Katsonis , E. Lacaze , A. Ferrarini , J. Mater. Chem. 2012, 22, 7088.

[119] J. Li , S. Xie , J. Meng , Y. Liu , Q. Zhan , Y. Zhang , L. Shui , G. Zhou , B. L. Feringa , J. Chen , CCS Chem. 2024, 6, 427–438.

[120] M. Urbanski , C. G. Reyes , J. Noh , A. Sharma , Y. Geng , V. Subba Rao Jampani , J. P. F. Lagerwall , J. Phys. Condens. Matter 2017, 29, 133003. 28199222 10.1088/1361-648X/aa5706

[121] J. Fan , Y. Li , H. K. Bisoyi , R. S. Zola , D. Yang , T. J. Bunning , D. A. Weitz , Q. Li , Angew. Chem. 2015, 127, 2188–2192. 10.1002/anie.201410788 25487252

[122] T. Orlova , S. J. Aßhoff , T. Yamaguchi , N. Katsonis , E. Brasselet , Nat. Commun. 2015, 6, 7603. 26145716 10.1038/ncomms8603 PMC4506501

