# Supplementary material for: A Multiresponsive Ferrocene‐Based Chiral Overcrowded Alkene Twisting Liquid Crystals
Source: Angew Chem Int Ed Engl. 2024 Nov 6;64(1):e202413047. doi: 10.1002/anie.202413047 (PMC11701369; doi:10.1002/anie.202413047)
Supplement: Supplementary file 2 — Supporting Information [file ANIE-64-e202413047-s001.pdf]

Angewandte Chemie
Eine Zeitschrift der Gesellschaft Deutscher Chemiker

Supporting Information

A Multiresponsive Ferrocene-Based Chiral Overcrowded Alkene Twisting Liquid Crystals

*M. Fellert, R. Hein, A. Ryabchun, Y. Gisbert, C. N. Stindt, B. L. Feringa**

Supporting Information

A Multiresponsive Ferrocene-Based Chiral Overcrowded Alkene Twisting Liquid Crystals

Maximilian Fellert,[+] Robert Hein,[+] Alexander Ryabchun, Yohan Gisbert, Charlotte N. Stindt, Ben L. Feringa*

Stratingh Institute for Chemistry, University of Groningen, Nijenborgh 3, 9747AG Groningen (The Netherlands)
b.l.feringa@rug.nl

Table of contents

1. Supplementary Experimental Procedures S2
   1.1. General Methods S2
   1.2. Computational Methods S3
   1.3. X-ray Analysis S3
   1.4. Synthetic Procedures S4
2. X-ray Structural Data S8
3. DFT Calculations S9
4. Structural Analysis S13
5. Thermal and Photoswitching S14
6. CD Spectra S18
7. (Spectro)electrochemistry and Redox-Switching S19
8. Liquid Crystal (LC) Experiments S22
9. HPLC Chromatograms S27
10. NMR Spectra of New Compounds S30
11. References S35

1. Supplementary Experimental Procedures

1.1. General Methods

Commercial reagents and solvents: All chemicals and solvents were purchased from commercial suppliers unless otherwise stated. Anhydrous solvents were obtained using a MBraun SPS 800 system and stored under N_2. $TBAPF_6$ was of electrochemical grade and obtained from Sigma Aldrich.

Synthesized reagents: FcPrec, *(S)*-**FcKetone and** *(R)*-**FcKetone** were synthesized according to reported literature procedures[1,2] and characterized using standard characterization techniques.

Synthesis and purification: Standard Schlenk techniques were used, employing nitrogen or argon as the inert gas. If they were not performed at room temperature, the reaction temperatures refer to the temperature of the heating/cooling bath or heating block.

Flash column chromatography was performed on a Biotage Selekt system using the indicated solvents. TLC analysis was done on Merck silica gel 60 F_{254} aluminum sheets, and compounds were visualized with a UV lamp (254 nm or 365 nm).

NMR:

Full characterization of the newly synthesized compounds (including ^{1}H, ^{13}C, and 2D NMR experiments) was performed using a Bruker Avance Neo 600 (600 MHz), a Varian Mercury Plus (400 MHz) or Agilent MR (400 MHz) spectrometer. Chemical shifts (δ) are given in parts per million (ppm) relative to TMS, using the solvent residual peak as internal standard ($CDCl_3$: δ = 7.26 for ^{1}H, δ = 77.16 for ^{13}C; toluene-d_8: δ = 2.08 for ^{1}H, δ = 20.43). Data is reported as follows: chemical shifts (δ) in ppm, multiplicity (s = singlet, d = doublet, t = triplet, m = multiplet), coupling constants *J* (Hz), and integration. Variable-temperature NMR and in-situ irradiation experiments were performed using a Varian Inova 500 (500 MHz) spectrometer. NMR irradiation experiments were performed at the indicated temperature with a fiber-coupled LED and a 1500 µm optical fiber (FT1500UMT) to guide the light directly into the NMR tube inside the NMR spectrometer. Relaxation experiments at various temperatures were performed by equilibrating the sample at the given temperature, tuning, locking, and shimming the NMR, irradiating until a stationary state was reached, stopping the irradiation, waiting until the lock signal stabilized and finally starting to record the decay.

High-resolution mass (HMRS) spectra were recorded on a Thermofisher LTQ Orbitrap XL.

HPLC analysis was performed using a Shimadzu SPD M10AVP diode array detector using Chiralcel columns with mixtures of HPLC-grade *n*-heptane and 2-propanol as the eluent and a column temperature of 40 °C.

UV-vis absorption spectra were recorded on a Agilent Cary 8454 spectrophotometer in 1 cm quartz cuvettes.

CD spectra were recorded on a Jasco J-815 spectropolarimeter. The LEDs were attached via a 1500 µm optical fiber (M93L01).

Irradiation experiments were performed using fiber-coupled LEDs (M365F1, M420F1) powered with a T-Cube™ LEDD18 driver obtained from Thorlabs Inc.

Electrochemical Measurements were carried out with a Palmsense 4 potentiostat and a three-electrode setup comprising a glassy carbon disk working electrode (3 mm diameter), a Pt wire counter electrode and a non-aqueous Ag/$AgNO_3$ reference electrode (10 mM $AgNO_3$ in CH_3CN, 100 mM $TBAPF_6$). Prior to each experiment, the working electrode surface was polished using a 0.05 µM alumina slurry. All experiments were carried out in CH_2Cl_2 (HPLC grade) under ambient conditions and in the presence of oxygen. $TBAPF_6$ was used as supporting electrolyte throughout (100 mM for standard voltametric experiments and 200 mM for spectroelectrochemistry). CVs and SWVs were recorded with a step size of 2 mV. For the latter an amplitude of 20 mV and a frequency of 20 Hz were employed. All potentials are reported with respect to Fc/Fc^+.

Spectroelectrochemical Experiments were carried out using a 1 mm pathlength spectroelectrochemical cuvette (ALS Japan) with a Pt mesh electrode, a non-aqueous Ag/$AgNO_3$ reference electrode (10 mM $AgNO_3$ in CH_3CN, 100 mM $TBAPF_6$) and a Pt wire counter electrode. A 320 nm cutoff filter was used to minimize UV exposure. To ensure complete electrolysis, potentials that were at least 150 mV anodic or cathodic of the respective redox waves were applied to generate **FcD^{2+}** or **FcD**, respectively. To generate **FcD^{+}**, the potential exactly in the middle of both redox waves was applied. UV-vis spectra were recorded every 15 s, typically for 10-15 min per cycle.

1.2. Computational Methods

Starting from the crystal structure obtained for (*R*,*R*)-(*M*,*M*)-*E*-syn-**FcD**, a series of low-lying conformers were generated using CREST[3–5] (Conformer–Rotamer Ensemble Sampling Tool) at the GFN2-xTB[6] level of theory. These conformers were further studied using the Orca 5.0.4 package.[7] Geometries were optimized with the composite method r^2SCAN-3c,[8] using the conductor-like polarizable continuum CPCM(Toluene) solvent model.[9] Only four low-lying conformers (*P* and *M* helicities of both *E* and *Z* isomers) were found for the neutral and mono-cationic states. The thermochemical data were calculated at –80, 25, 90, and 100 °C at the same level of theory. The minima and transition states had no or one imaginary frequency, respectively.

1.3. X-ray Analysis

Crystals of *E-syn*-**FcD** were obtained by slow evaporation from a concentrated solution in ethyl acetate. A single crystal was mounted on a cryoloop and analyzed on a Bruker-AXS D8

Venture diffractometer, using MoKα radiation (λ = 0.71073 Å). The data collection was done at room temperature under ambient conditions. The Bruker APEX4 software suite was used for data collection and processing, and a multi-scan absorption correction was applied using SADABS (SADABS-2016/2).[10] The structure was solved using SHELXT.[11] Subsequent refinement was done using SHELXL[12] in the OLEX2 software package.[13] Hydrogen atoms were generated by geometrical considerations and refined using a riding model. No A- or B-level alerts were raised by CheckCIF for the fully refined structure.

Deposition Number 2369530 contains the supplementary crystallographic data for this paper. These data can be obtained free of charge by the Cambridge Crystallographic Data Centre https://www.ccdc.cam.ac.uk/structures/.

1.4. Synthetic Procedures

FcPrec

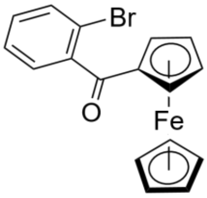

Ferrocene (3.391 g, 18.27 mmol, 1.0 equiv.) was dissolved in CH_2Cl_2 (20 mL), the solution was cooled to 0 °C, and a solution of $AlCl_3$ (2.430 g, 18.27 mmol, 1.0 equiv.) and 2-bromobenzoyl chloride (2.40 mL, 18.27 mmol, 1.0 equiv.) in CH_2Cl_2 (20 mL) was added dropwise. The reaction mixture was warmed to room temperature and stirring was continued for 2 h. After the addition of ice, the mixture was extracted with CH_2Cl_2 three times, washed with brine, dried over Na_2SO_4, filtered, and concentrated *in vacuo*. Purification by flash column chromatography on SiO_2 (pentane: EtOAc 1:0 to 4:1) afforded **FcPrec** as a red solid (5.260 g, 14.25 mmol, 78%).

The NMR data match those reported in the literature.[2]

(S)-**FcKetone**

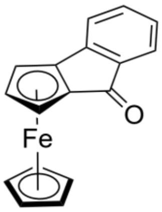

$Pd(OAc)_2$ (15.2 mg, 0.0677 mmol, 0.05 equiv.), Cs_2CO_3 (0.883 g, 2.710 mmol, 2.0 equiv.) and (*S*)-BINAP (88.6 mg, 0.142 mmol, 0.1 equiv.) were transferred into an oven-dried Schlenk flask and suspended in toluene (6 mL). After stirring for 10 min at room temperature, **FcPrec** (0.502 g, 1.355 mmol, 1.0 equiv.) was added, and the flask was placed in an oil bath preheated to 100 °C. The reaction mixture was stirred at 100 °C for 16 h. After cooling to room temperature, the reaction mixture was filtered over a plug of silica and eluted with CH_2Cl_2. Column chromatography on SiO_2 (pentane/CH_2Cl_2 1:0 → 0:1) afforded the desired product as a red solid (0.279 g, 0.968 mmol, 72%).

The NMR data match those reported in the literature.[2]

HPLC (Chiralcel OD-H, *n*-heptane/2-propanol 97:3, 1.0 mL/min): retention times (min) 10.11 ((*S*)-**FcKetone**, major), 14.19 ((*R*)-**FcKetone**, minor), *ee* = 99%.

(R)-**FcKetone** (0.301 g, 1.05 mmol, 77%) was prepared following the same procedure using (*R*)-BINAP.

HPLC (Chiralcel OD-H, *n*-heptane/2-propanol 97:3, 1.0 mL/min): retention times (min), 10.19 ((*S*)-**FcKetone**, minor), 13.51 ((*R*)-**FcKetone**, major), *ee* = 99%.

<u>(*S*)-**FcThioketone**</u>

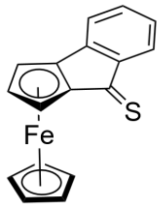

(*S*)-**FcKetone** (0.250 g, 0.868 mmol, 1.0 equiv.) was dissolved in THF (6 mL) and Lawesson's reagent (1.404 g, 3.471 mmol, 4.0 equiv.) was added. The reaction mixture was heated to 65 °C for 1.5 h. After cooling to room temperature, the solvent was reduced in vacuo and the crude product was purified by column chromatography on SiO_2 (pentane:CH_2Cl_2 9:1 to 4:1) to afford (*S*)-**FcThioketone** as a blue-green solid (0.257 g, 0.845 mmol, 97%).

^{1}H NMR (400 MHz, $CDCl_3$) δ = 7.59 (m, 1H), 7.33 – 7.25 (m, 1H), 7.10 – 6.99 (m, 2H), 5.32 – 5.27 (m, 1H), 5.22 – 5.16 (m, 1H), 5.06 – 5.01 (m, 1H), 4.08 (s, 5H).

$^{13}C\{^1H\}$ NMR (101 MHz, $CDCl_3$) δ = 226.7, 147.4, 141.9, 132.8, 126.7, 122.9, 119.9, 91.4, 91.2, 77.0, 75.8, 67.5, 67.4.
HRMS (ESI+): calculated for $[C_{17}H_{12}FeS + H]^+$ 304.0004, found 340.0001.

(*R*)-**FcThioketone** (0.216 g, 0.710 mmol, 89%) was prepared following the same procedure using (*R*)-**FcKetone**.

<u>(*S*,*S*)-(*P*,*P*)-*E*-*syn*-**FcD**</u>

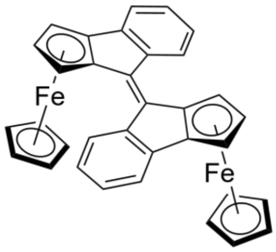

(S)-**FcThioketone** (0.130 g, 0.427 mmol, 1.0 equiv.) was dissolved in THF (16 mL). 8 mL of this solution were cooled to –20 °C and a 2 M solution of trimethylsilyldiazomethane in hexane (0.12 mL, 0.235 mmol, 0.55 equiv.) was added. The reaction mixture was warmed to room temperature, the remaining 8 mL of the solution of (*S*)-**FcThioketone** in THF were added and stirring was continued for 15 min. After addition of TBAF (0.86 mL, 0.855 mmol, 2.0 equiv.), the reaction mixture was stirred for 30 min. The solvents were removed *in vacuo* and the crude product was purified by column chromatography on SiO_2 (pentane:CH_2Cl_2 1:0 to 3:1) to afford (*S*,*S*)-(*P*,*P*)-*E*-*syn*-**FcD** as a dark purple solid (0.089 g, 0.164 mmol, 77%).

^1H NMR (400 MHz, $CDCl_3$) δ = 8.57 – 8.50 (m, 2H), 7.45 – 7.38 (m, 2H), 7.35 – 7.22 (m, 4H), 4.96 – 4.90 (m, 2H), 4.88 – 4.80 (m, 2H), 4.59 – 4.51 (m, 2H), 4.13 (s, 10H).
$^{13}C\{^1H\}$ NMR (101 MHz, $CDCl_3$) δ = 143.5, 140.6, 133.3, 127.6, 125.5, 125.2, 120.7, 91.9, 83.3, 72.5, 71.1, 64.1, 61.7.
HRMS (ESI+): calculated for $[C_{34}H_{24}Fe_2 + H]^+$ 544.0571, found 544.0574.
HPLC (Chiralcel OD-H, *n*-heptane/2-propanol 95:5, 1.0 mL/min): retention times (min) 5.84 ((*R*,*R*)-(*M*,*M*)-*E*-*syn*-**FcD**, minor), 10.38 ((*S*,*S*)-(*P*,*P*)-*E*-*syn*-**FcD**, major), *ee* = 99%.

(*R*,*R*)-(*M*,*M*)-*E*-*syn*-**FcD** (0.076 g, 0.140 mmol, 65%) was prepared following the same procedure using (*R*)-**FcThioketone**.

HPLC (Chiralcel OD-H, *n*-heptane/2-propanol 95:5, 1.0 mL/min): retention times (min) 6.01 ((*R*,*R*)-(*M*,*M*)-*E*-*syn*-**FcD**, major), 10.25 ((*S*,*S*)-(*P*,*P*)-*E*-*syn*-**FcD**, minor), *ee* = 99%.

(*R*,*R*)-(*M*,*M*)-*Z*-*anti*-**FcD**

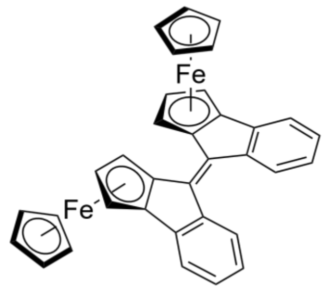

(*R*,*R*)-(*M*,*M*)-*E*-*syn*-**FcD** (15.0 mg, 49.3 µmol) was dissolved in dry toluene (5 mL) and heated to 100 °C for three days. After cooling to room temperature, the reaction mixture was purified by preparative TLC on SiO_2 (pentane:toluene:diethyl ether 98:1:1) to yield the desired (*R*,*R*)-(*M*,*M*)-*Z*-*anti*-**FcD** as a blue solid (2.05 mg, 6.74 µmol, 14%).

^{1}H NMR 600 MHz, $CDCl_3$) δ = 8.14 (d, J = 7.9 Hz, 2H), 7.39 (d, J = 7.5 Hz, 2H), 7.22 – 7.17 (m, 2H), 7.15 – 7.07 (m, 2H), 5.34 (d, J = 2.4 Hz, 2H), 4.85 (d, J = 2.2 Hz, 2H), 4.69 – 4.62 (m, 2H), 4.00 (s, 10H).

Due to its limited stability and fast backswitching, (*R*,*R*)-(*M*,*M*)-*Z*-*anti*-**FcD** could only be characterized by ^{1}H NMR spectroscopy.

2. X-ray Structural Data

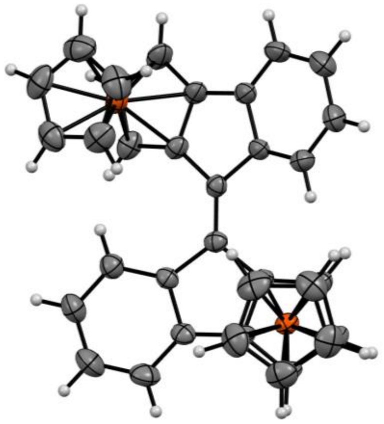

Table S1. Crystallographic data for (*R*,*R*)-(*M*,*M*)-*E*-*syn*-**FcD**

Empirical formula	$C_{34}H_{24}Fe_2$
Formula weight	544.23
Temperature/K	293
Crystal system	orthorhombic
Space group	$P2_12_12_1$
a/Å	14.6115(11)
b/Å	14.7451(12)
c/Å	22.6126(18)
α/°	90
β/°	90
γ/°	90
Volume/Å^3	4871.8(7)
Z	8
ρ_{calc}g/cm^3	1.484
μ/mm^{-1}	1.213
F(000)	2240.0
Crystal size/mm^3	0.476 × 0.394 × 0.055
Radiation	MoKα (λ = 0.71073)
2Θ range for data collection/°	5.526 to 59.2
Index ranges	-20 ≤ h ≤ 20, -20 ≤ k ≤ 20, -31 ≤ l ≤ 31
Reflections collected	288290
Independent reflections	13673 [R_{int} = 0.1408, R_{sigma} = 0.0434]
Data/restraints/parameters	13673/0/649
Goodness-of-fit on F^2	1.101
Final R indexes [I>=2σ (I)]	R_1 = 0.0431, wR_2 = 0.0759
Final R indexes [all data]	R_1 = 0.0660, wR_2 = 0.0857
Largest diff. peak/hole / e Å^{-3}	0.41/-0.31
Flack parameter	0.007(7)

Thermal ellipsoids in the ORTEP image are drawn at 50% probability. One molecule is shown, as the 2nd molecule was omitted for clarity.

3. DFT Calculations

Table S2. Summary of the Gibbs free energy differences for the energy minima and transition states of the isomers of **FcD** and **FcD$^+$** involved in the switching process, calculated at the r^{2}SCAN-3c/CPCM(toluene) level of theory. Boltzmann weight (BW) was evaluated for each minimum at the various temperatures employed for the experimental study. The Boltzmann distributions were calculated for isolated *E*/*Z* populations (in the absence of *E/Z* isomerization) and for the overall population (if *E/Z* isomerization is possible). For the cationic state, the counter anion was not considered.

	**25 °C**			**90 °C**		**100 °C**		**–80 °C**	
	**ΔG (kJ/mol)**	**BW (*E*/*Z*)**	**BW (all)**	**BW (*E*/*Z*)**	**BW (all)**	**BW (*E*/*Z*)**	**BW (all)**	**BW (*E*/*Z*)**	**BW (all)**
(*M*,*M*)-(*R*,*R*)-*E*-**FcD**	0.00	0.98	0.76	0.97	0.69	0.96	0.68	1.00	0.90
(*P*,*P*)-(*R*,*R*)-*E*-**FcD**	10.16	0.02	0.01	0.03	0.02	0.04	0.03	0.00	0.00
TS: THI-*E*-**FcD**	37.68								
(*M*,*M*)-(*R*,*R*)-*Z*-**FcD**	5.20	0.41	0.09	0.42	0.12	0.43	0.13	0.36	0.04
(*P*,*P*)-(*R*,*R*)-*Z*-**FcD**	4.26	0.59	0.14	0.58	0.17	0.57	0.17	0.64	0.06
TS: THI-*Z*-**FcD**	42.41								
(*M*,*M*)-(*R*,*R*)-*E*-**FcD$^+$**	1.44	0.98	0.34	0.96	0.35	0.96	0.35	1.00	0.29
(*P*,*P*)-(*R*,*R*)-*E*-**FcD$^+$**	11.06	0.02	0.01	0.04	0.01	0.04	0.02	0.00	0.00
TS: THI-*E*-**FcD$^+$**	40.13								
(*M*,*M*)-(*R*,*R*)-*Z*-**FcD$^+$**	6.40	0.07	0.05	0.11	0.07	0.11	0.07	0.02	0.01
(*P*,*P*)-(*R*,*R*)-*Z*-**FcD$^+$**	0.00	0.93	0.61	0.89	0.57	0.89	0.56	0.98	0.70
TS: THI-*Z*-**FcD$^+$**	41.65								

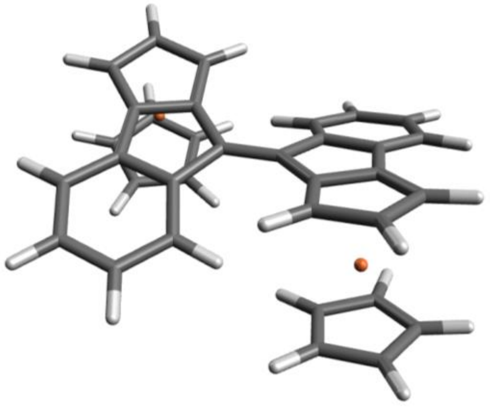

Figure S1. Calculated structure of (*M*,*M*)-(*R*,*R*)-*E*-**FcD** optimized at the r^{2}SCAN-3c/CPCM(toluene) level of theory.

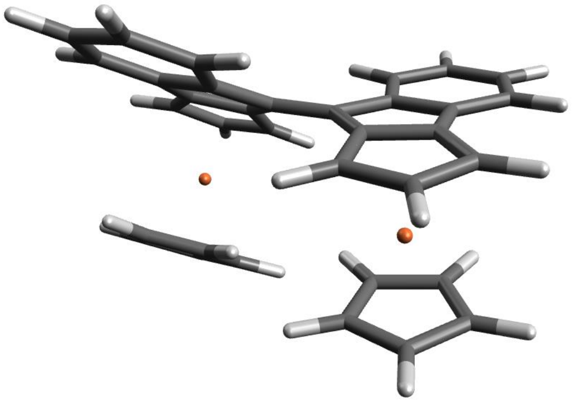

Figure S2. Calculated structure of (*P*,*P*)-(*R*,*R*)-*E*-**FcD** optimized at the r^2SCAN-3c/CPCM(toluene) level of theory.

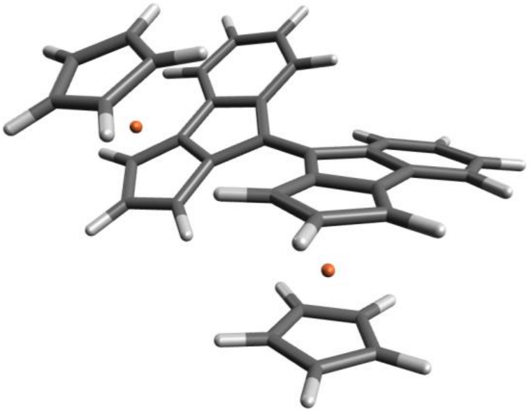

Figure S3. Calculated structure of (*M*,*M*)-(*R*,*R*)-*Z*-**FcD** optimized at the r^2SCAN-3c/CPCM(toluene) level of theory.

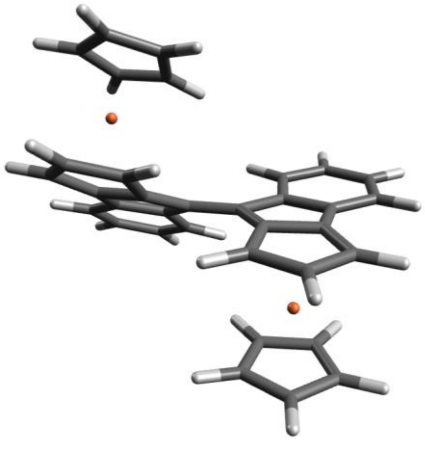

Figure S4. Calculated structure of (*P*,*P*)-(*R*,*R*)-*Z*-**FcD** optimized at the r^2SCAN-3c/CPCM(toluene) level of theory.

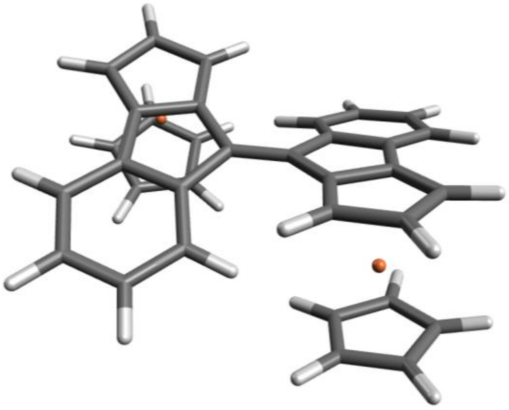

Figure S5. Calculated structure of (*M*,*M*)-(*R*,*R*)-*E*-**FcD⁺** optimized at the r^{2}SCAN-3c/CPCM(toluene) level of theory.

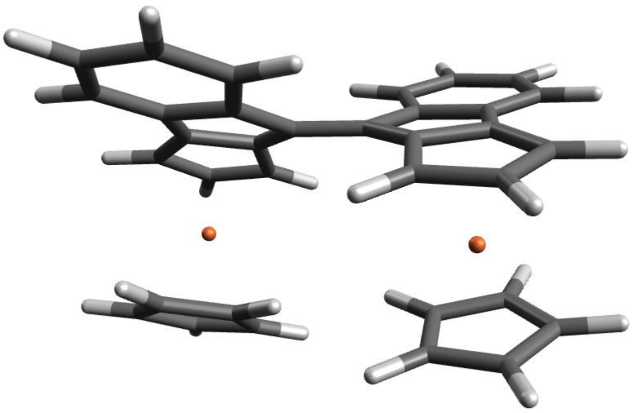

Figure S6. Calculated structure of (*P*,*P*)-(*R*,*R*)-*E*-**FcD⁺** optimized at the r^{2}SCAN-3c/CPCM(toluene) level of theory.

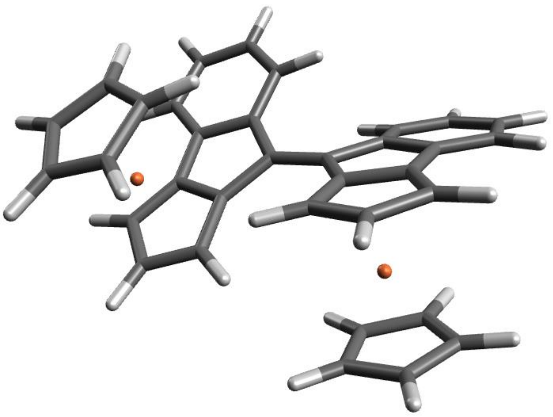

Figure S7. Calculated structure of (*M*,*M*)-(*R*,*R*)-*Z*-**FcD⁺** optimized at the r^{2}SCAN-3c/CPCM(toluene) level of theory.

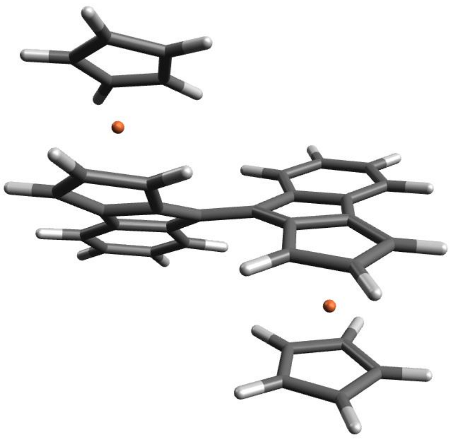

Figure S8. Calculated structure of (*P*,*P*)-(*R*,*R*)-*Z*-**FcD**$^{+}$ optimized at the r^{2}SCAN-3c/CPCM(toluene) level of theory.

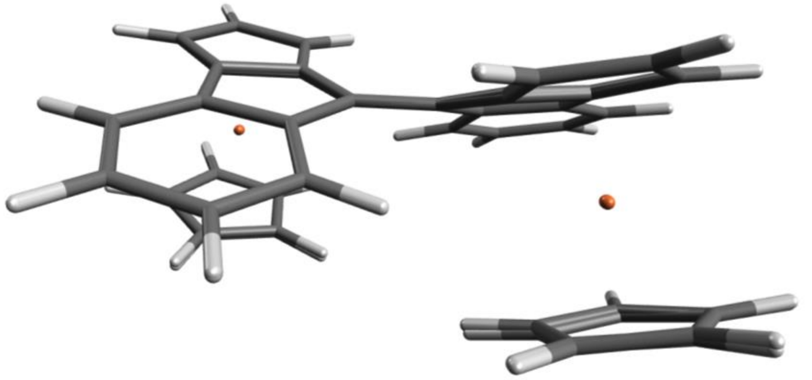

Figure S9. Overlay of the calculated structures of (*M*,*M*)-(*R*,*R*)-*E*-**FcD** and oxidized (*M*,*M*)-(*R*,*R*)-*E*-**FcD**$^{+}$ optimized at the r^{2}SCAN-3c/CPCM(toluene) level of theory. Very minor structural differences can be observed, such as a displacement of the Cp rings and a slight twisting around the central axis.

4. Structural Analysis

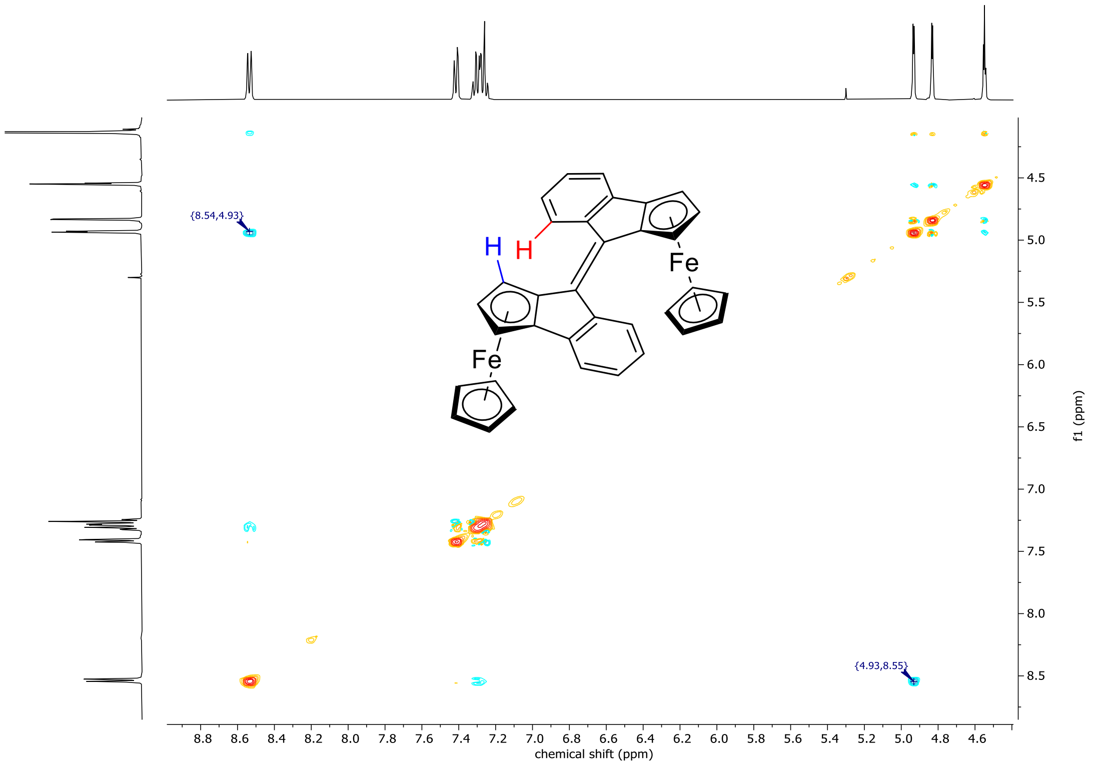

Figure S10. Zoom-in of the NOESY ^{1}H NMR spectrum of *E*-*syn*-**FcD** (600 MHz, $CDCl_3$, 25 °C). The signal corresponding to the NOE between the ferrocene-bound (blue, 4.93 ppm) and aromatic (red, 8.54 ppm) proton is characteristic for the *E*-*syn*-isomer of **FcD**.

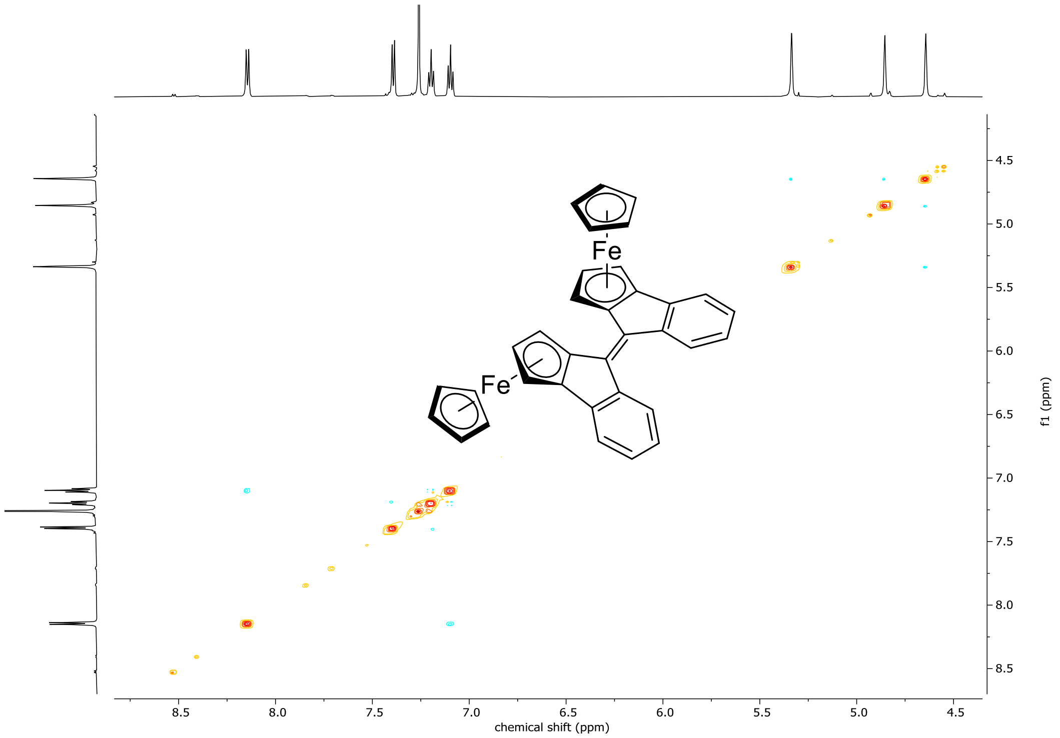

Figure S11. Zoom-in of the NOESY ^{1}H NMR spectrum of *Z*-*anti*-**FcD** (600 MHz, $CDCl_3$, 25 °C). The absence of a signal corresponding to a NOE between a ferrocene-bound and an aromatic proton is characteristic for the *Z*-*anti*-isomer of **FcD**.

5. Thermal and Photoswitching

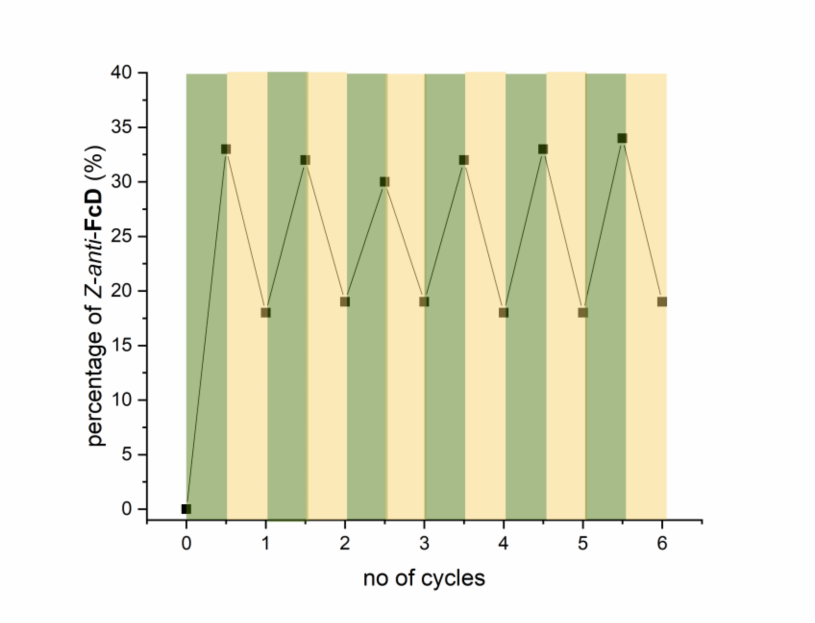

Figure S12. Fatigue study of *E/Z*-switching of **FcD**, measured by ^{1}H NMR spectroscopy (toluene-d_8) starting from pure *E*-*syn*-**FcD**. Changes in the integrals of the doublets at 8.53 (*E*-*syn*-**FcD**) and 8.14 ppm (*Z*-*anti*-**FcD**), plotted as the percentage of *Z*-*anti*-**FcD** in the overall mixture, were monitored upon heating to 100 °C for 48 h (green shaded areas), cooling to room temperature, irradiating with 595 nm light for 10 min (yellow shaded areas), and repeating the cycle.

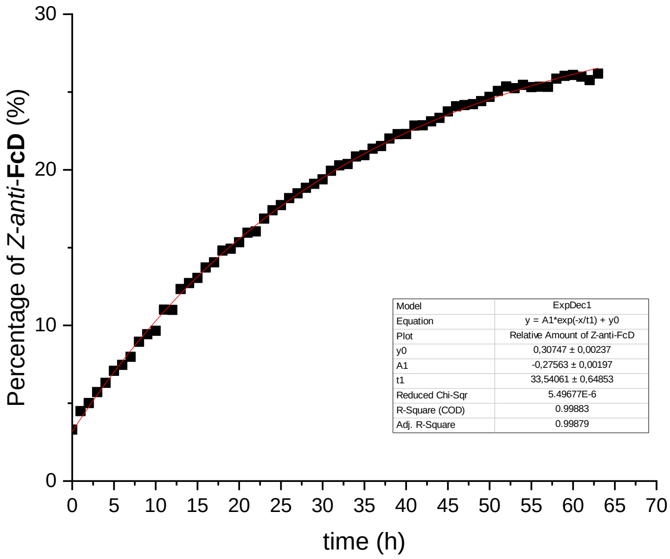

Figure S13. Thermal switching of *E*-*syn*-**FcD** at 90 °C, measured by ^{1}H NMR spectroscopy (toluene-d_8). Changes in the integral of the doublet 8.14 ppm (*Z*-*anti*-**FcD**) were monitored and plotted as the percentage of Z-anti-**FcD** in the overall mixture. An exponential fit to the curve allows for the determination of the asymptotic plateau (y_0), indicating an E:Z ratio of 69:31, as well as the determination of the thermal switching half-life time $t_{1/2,\ 90\ °C} = t_1 * \ln(2) = 23$ h.

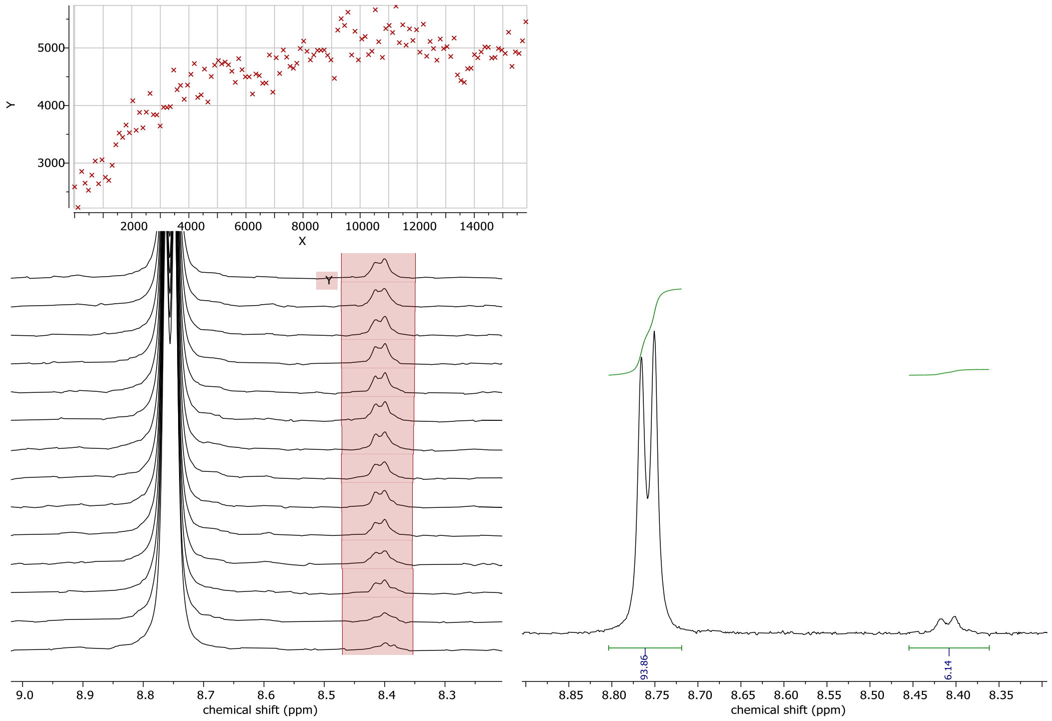

Figure S14. ^{1}H NMR (500 MHz, toluene-d$_8$, –80 °C) monitoring of the conversion of *E*-**FcD** into *Z*-**FcD** upon *in situ* irradiation with 595 nm UV light (left) providing a steady state composition with a 94:6 *E*:*Z* isomeric ratio (right).

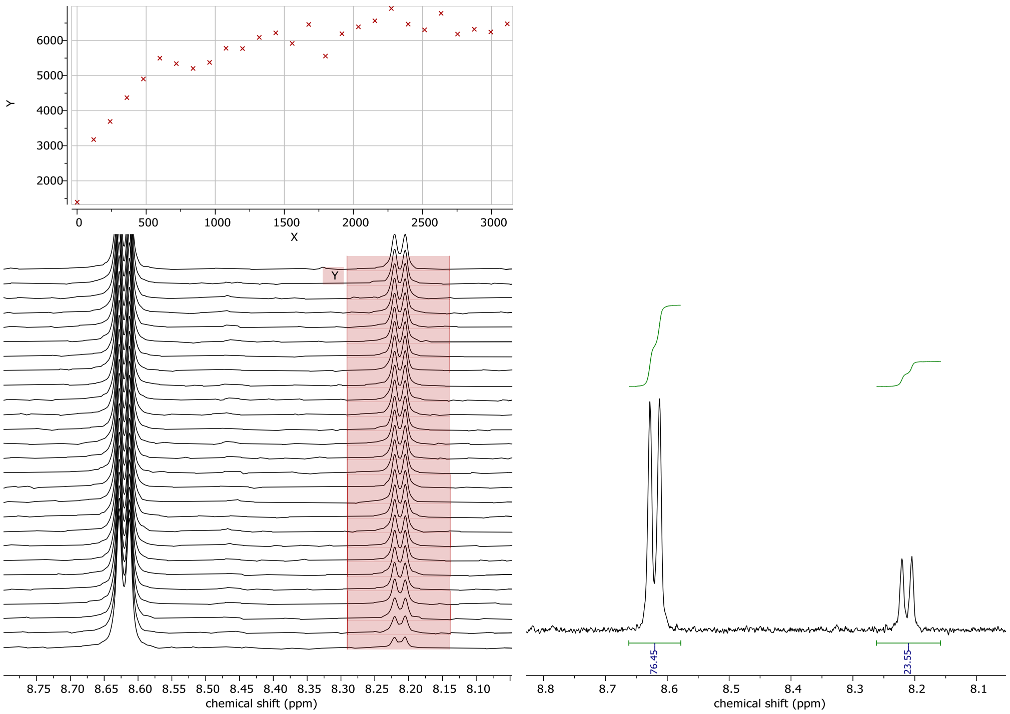

Figure S15. ^{1}H NMR (500 MHz, toluene-d$_8$, 90 °C) monitoring of the conversion of an *E*-**FcD/** *Z*-**FcD** mixture in a 94:6 ratio into *Z*-**FcD** upon *in situ* irradiation with 595 nm light (left) showing a steady state composed of a 76:24 *E*:*Z* isomeric ratio (right).

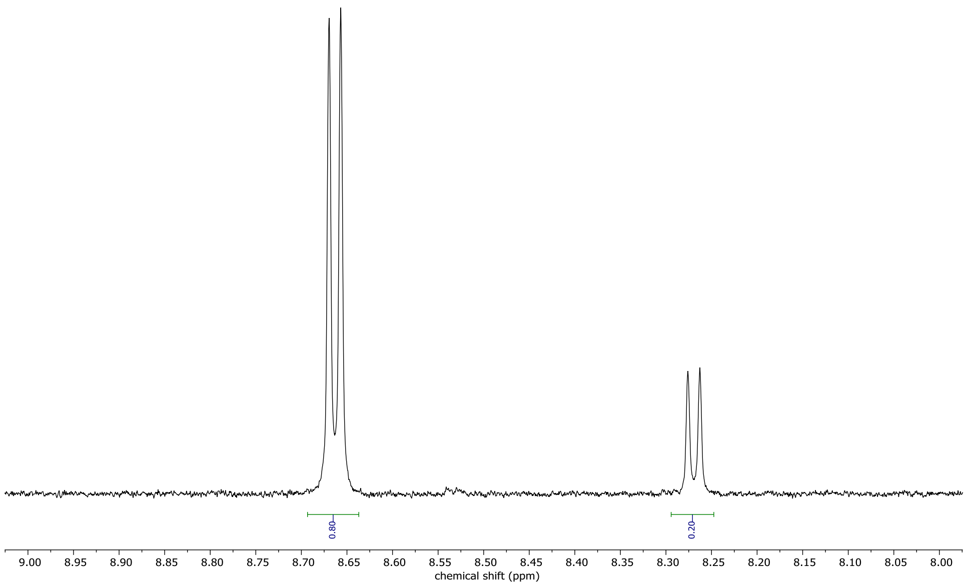

Figure S16. Zoom-in of the ^{1}H NMR spectrum of a mixture of *E*-*syn*-**FcD** and *Z*-*anti*-**FcD** (80:20) after irradiation of *E*-*syn*-**FcD** with 395 nm light for 10 min at 20 °C (600 MHz, toluene-d8, 25 °C).

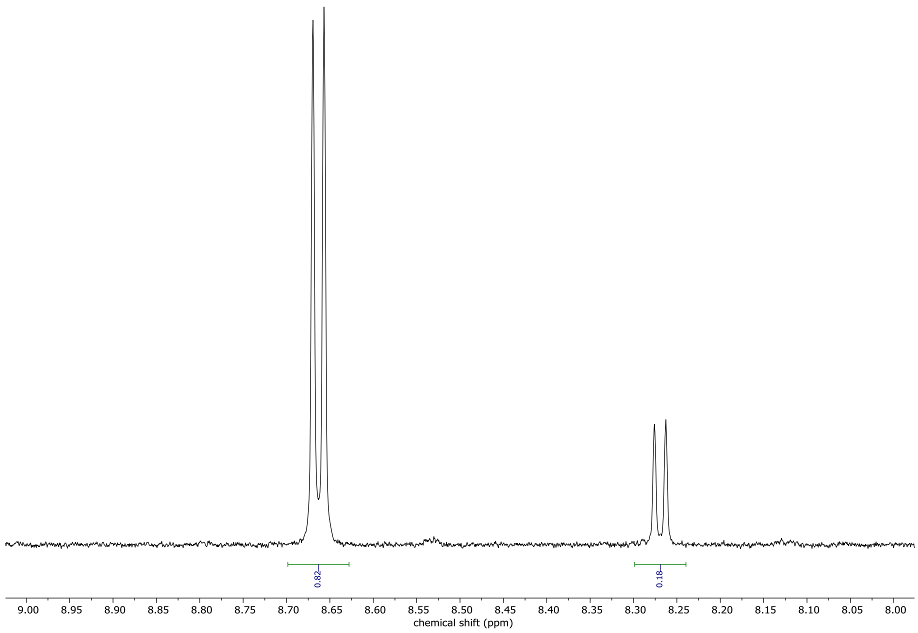

Figure S17. Zoom-in of the ^{1}H NMR spectrum of a mixture of *E*-*syn*-**FcD** and *Z*-*anti*-**FcD** (82:18) after irradiation of *E*-*syn*-FcD with 595 nm light for 10 min at 20 °C (600 MHz, toluene-d8, 25 °C).

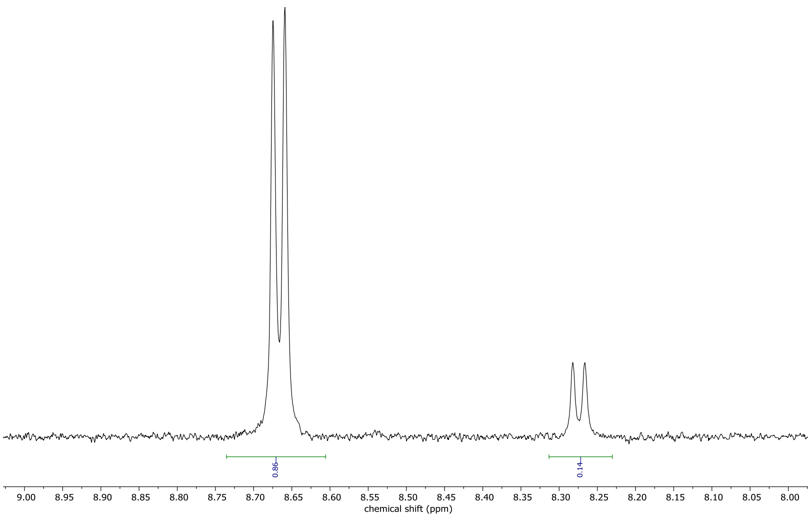

Figure S18. Zoom-in of the ^{1}H NMR spectrum of a mixture of *E*-*syn*-**FcD** and *Z*-*anti*-**FcD** (86:14) after irradiation of *E*-*syn*-**FcD** with 780 nm light for 10 min at 20 °C (500 MHz, toluene-d8, 25 °C).

6. CD Spectra

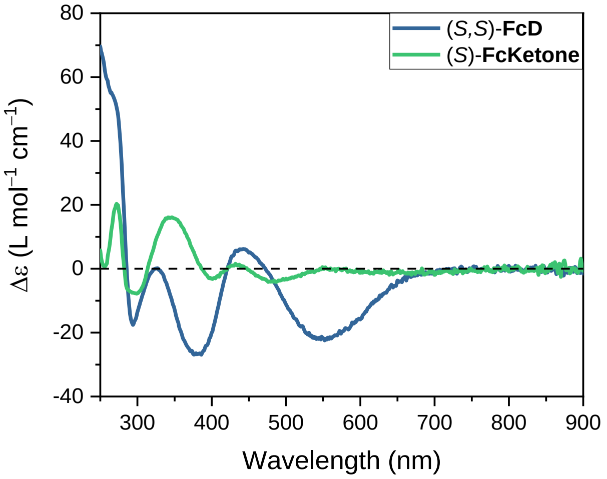

Figure S19. CD spectra of (*S*,*S*)-**FcD** and its (*S*)-**FcKetone** precursor in CH_2Cl_2.

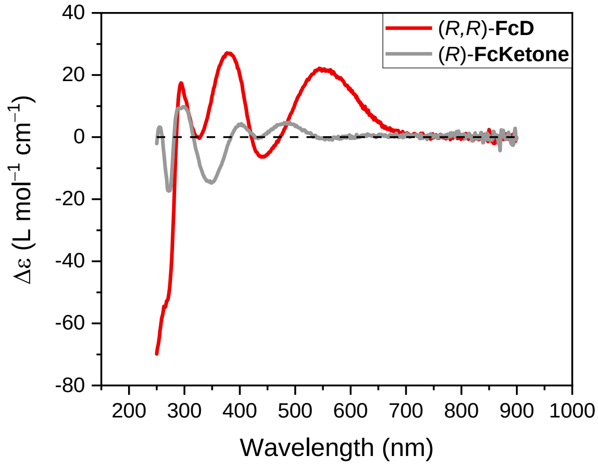

Figure S20. CD spectra of (*R*,*R*)-**FcD** and its (*R*)-**FcKetone** precursor in CH_2Cl_2.

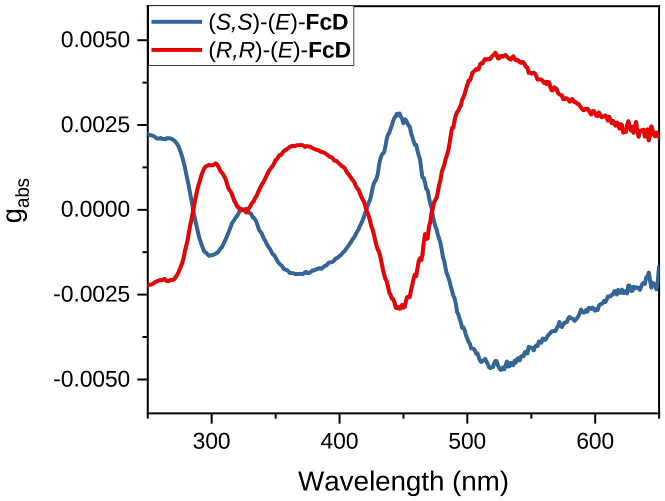

Figure S21. g-Factor plot (Δε/ε) for (*S*,*S*)-*E*-**FcD** and (*R*,*R*)-*E*-**FcD**.

7. (Spectro)electrochemistry and Redox-Switching

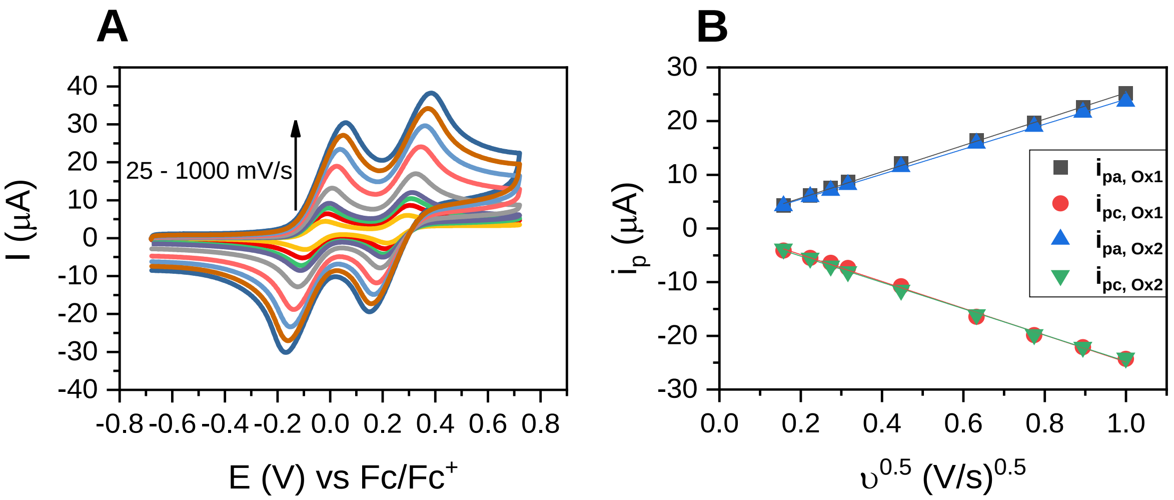

Figure S22. A) CVs of 500 µM (*R*,*R*)-**FcD** in CH_2Cl_2, 100 mM $TBAPF_6$ at varying scan rates. B) The associated anodic and cathodic peak currents as a function of the square-root of the scan rate including linear fits.

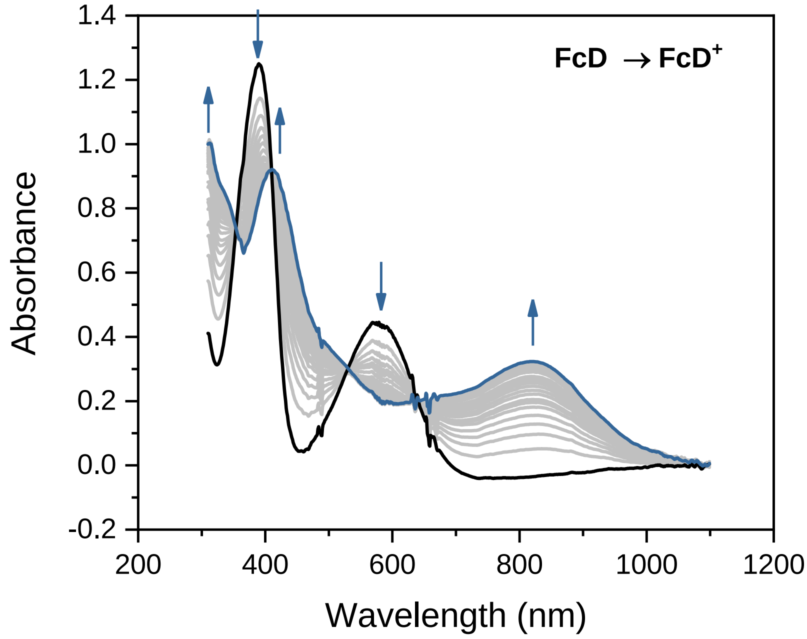

Figure S23. UV-vis spectra of the spectroelectrochemical conversion of (*R*,*R*)-*E*-**FcD** (black) to (*R*,*R*)-*E*-**FcD**$^+$ (blue) by electrolysis in CH_2Cl_2, 200 mM $TBAPF_6$. See Figure 4C for the changes in absorbance at selected wavelength over multiple cycles.

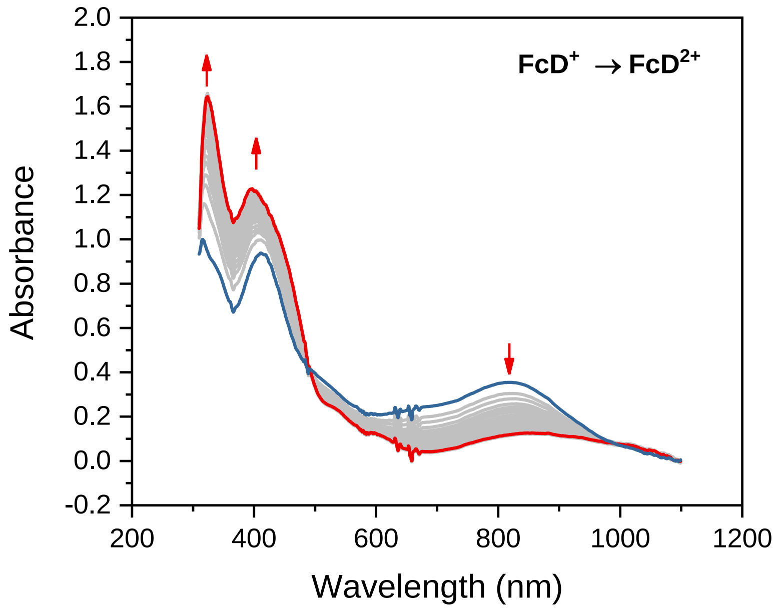

Figure S24. UV-vis spectra of the spectroelectrochemical conversion of (*R*,*R*)-*E*-**FcD** $^{+}$ (blue) to (*R*,*R*)-*E*-**FcD** $^{2+}$ (red) by electrolysis in CH_2Cl_2, 200 mM $TBAPF_6$. See Figure 4C for the changes in absorbance at selected wavelength over multiple cycles.

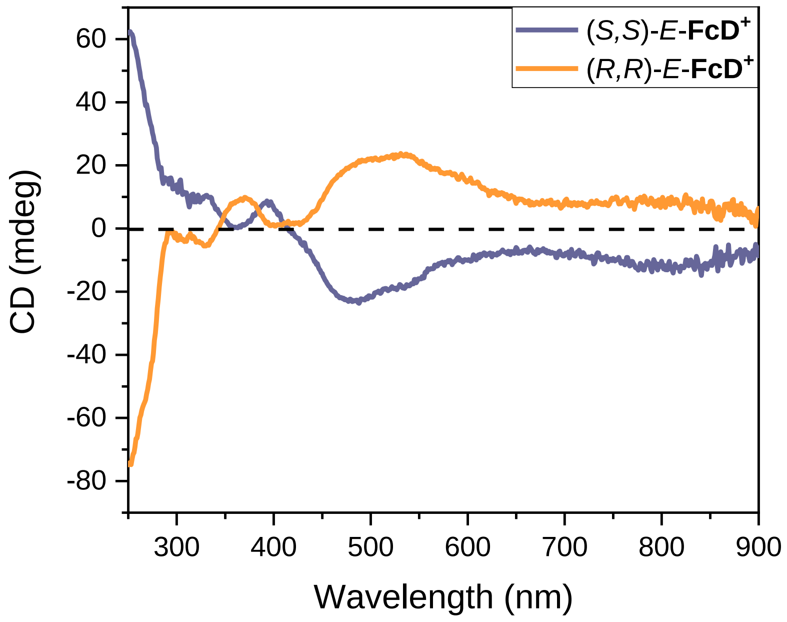

Figure S25. CD spectra of 50 µM (*S*,*S*)/(*R*,*R*)-*E*-**FcD**$^{+}$ generated by chemical oxidation with 1 equiv. magic blue (tris(4-bromophenyl)ammoniumyl hexachloroantimonate) in CH_2Cl_2.

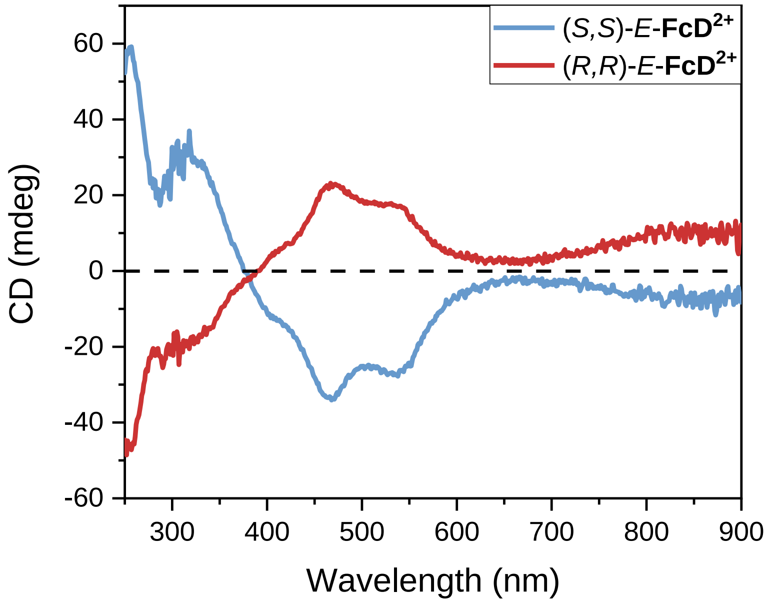

Figure S26. CD spectra of 50 µM (*S*,*S*)/(*R*,*R*)-*E*-**FcD**$^{2+}$ generated by chemical oxidation with 2 equiv. magic blue in CH_2Cl_2.

8. Liquid Crystal (LC) Experiments

Sample preparation

The doped LC mixtures were prepared by dissolving the necessary amounts of dopant(s) and nematic LC (E7) in dichloromethane, followed by solvent evaporation, and drying in vacuum. For helical twisting power (HTP) measurements mixtures containing 2 wt% of **FcKetone** or **FcD** were prepared, while 0.1 wt% mixtures were used for measuring the oxidized states of **FcD** due to their low solubility in LCs. These mixtures were introduced into wedge cells or θ-cells via capillarity. For experiments with droplets, two mixtures were utilized: i) a short pitch (few hundreds of nm) cholesteric mixture consisting of 4 wt% of (*R*,*R*)-*E*-**FcD** and 5 wt% (*R*)-BB ((*R*)-2,2'-methylenedioxy-1,1'-binaphthalene) dissolved in E7 LC, and ii) a long pitch (few µm) cholesteric mixture consisting of 2 wt% of (*R*,*R*)- *E*-**FcD** dissolved in E7. The HTP of (*R*)-BB in E7 is –61.2 as measured in a wedge cell. As a control, two mixtures with short and long cholesteric pitches, composed of 6 wt% and 0.5 wt% of (*R*)-BB, respectively, were employed. Chiral droplets were generated by vortexing the cholesteric mixture in 1 wt% poly(vinylalcohol) (Sigma-Aldrich) aqueous solution, followed by sonication for 30 s. Poly(vinylalcohol) was used to stabilize the emulsion and to promote planar LC alignment at droplet-water interface.

A supported cholesteric LC layer was prepared by placing a drop of material onto a transmission electron microscopy copper grid (300 mesh size, Sigma-Aldrich) on a microscopy glass slide. Excess material was removed with a paper tissue.

Optical LC measurements

Optical imaging and measurements were performed using a polarized optical microscope (POM) Eclipse LV100N-POL (Nikon, Japan) and Nis-Element-D software. The reflection spectra of the supported cholesteric layer were measured between crossed polarizers using a spectrometer HR2000+ (Ocean Optics) coupled to a polarized optical microscope BX51 (Olympus).

HTP and screw-sense measurements

HTPs of **FcD** and the ketone precursors were determined by the Cano method[14] using LC mixtures (E7) containing 2 wt% of dopant. The sign of the cholesteric helical structure was determined exploiting a rotatable analyzer as previously reported.[15] The wedge cells with $tan\theta$ = 0.0192 or 0.0423 were purchased from E.H.C. Co. Ltd. (Japan).

The Cano method utilizes a wedge cell with known opening angle θ, where both glass substrates promote unidirectional alignment parallel to each other. When cholesteric material is introduced to such confinement, the defect (disclination) lines appear in the contacts of areas with different number of half-turns of cholesteric helix due to strong anchoring at the interfaces

(Figure S27). The distance between two neighboring lines (*l*) is defined by the cholesteric pitch (p) according to Equation 1:

$$p = 2l \times \tan\theta \qquad (1)$$

The HTP of the chiral dopant can be estimated by the following Equation 2:

$$HTP = \pm(2l \times c \times \tan\theta)^{-1} \qquad (2)$$

where *c* is the concentration of the chiral dopant. Conventionally, the sign of the HTP is positive for right-handed cholesteric phase and negative for the left-handed one.

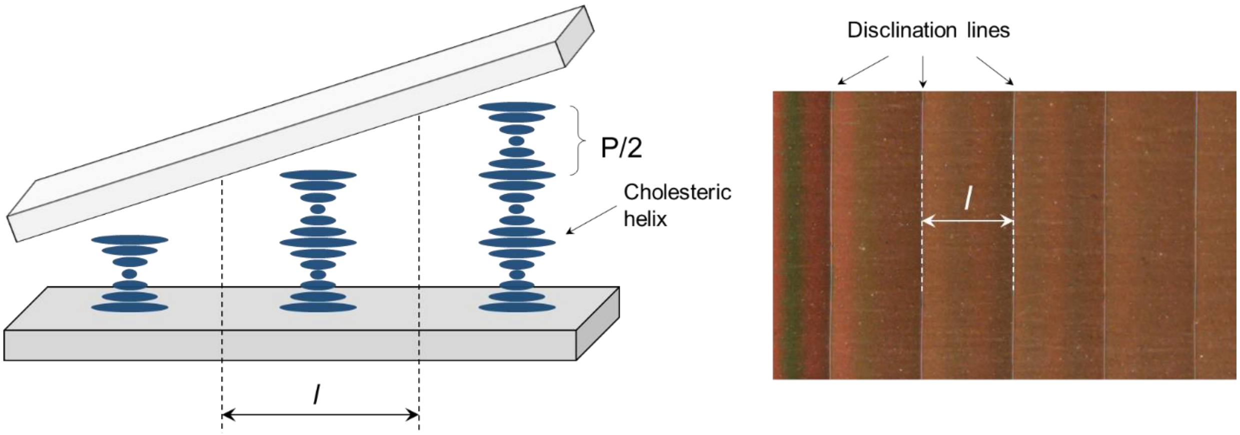

Figure S27. Schematic representation of the wedge cell filled with cholesteric LC and its typical texture observed by polarized optical microscopy (on the right).

The screw sense and HTP values of the **FcD⁺** (0.1 wt%) in LC were determined with a θ-cell that allows precise and reliable measurement of extremely large cholesteric helix pitch even in the centimeter range.[14,15] The details of the method and the θ-cell fabrication protocol described in a publication by Ryabchun et al.[16]

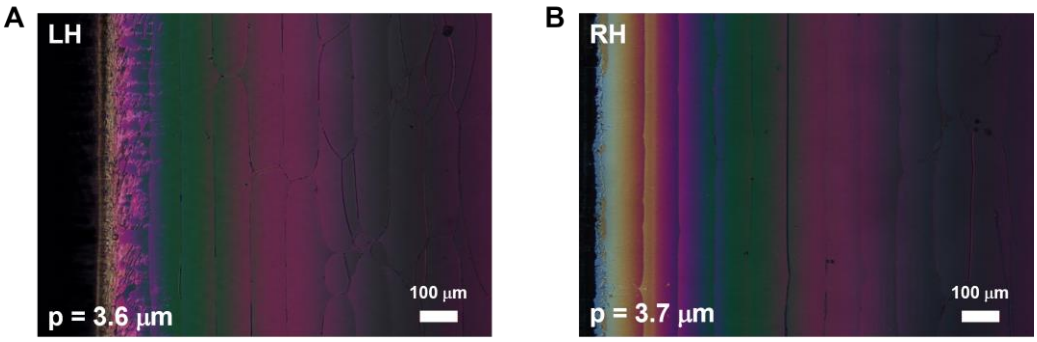

Figure S28. POM image of the wedge cell filled with (*R*,*R*)-*E*-**FcD** 2 wt% in E7 (a) and (*S*,*S*)-*E*-**FcD** 2 wt% in E7 (b). Wedge angle of the cell: tanθ=0.0192. LH and RH correspond to left- and right-handed cholesteric structure, respectively; p – cholesteric pitch.

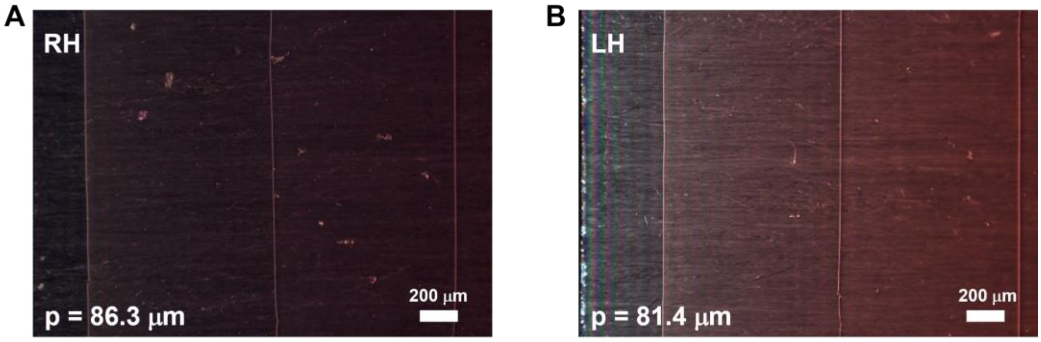

Figure S29. POM image of the wedge cell filled with (*R*)-**FcKetone** 2 wt% in E7 (A) and (*S*)-**FcKetone** 2 wt% in E7 (B). Wedge angle of the cell: tanθ=0.0423. LH and RH correspond to left- and right-handed cholesteric structure, respectively; p - cholesteric pitch.

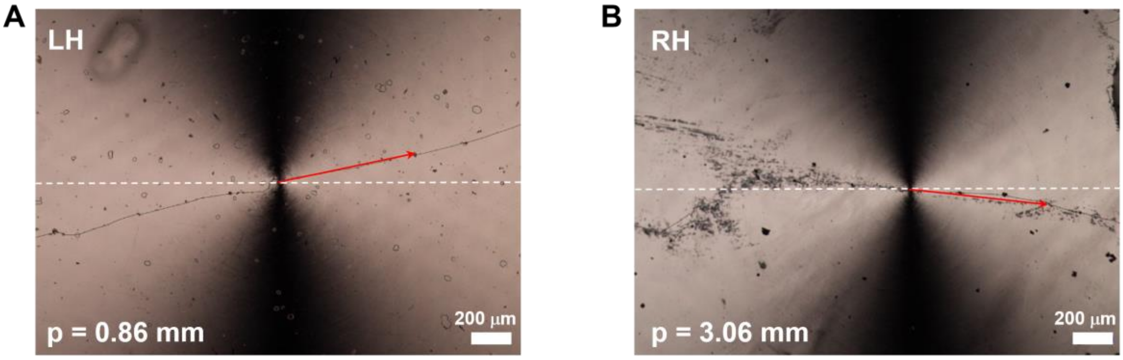

Figure S30. POM image of the θ-cell filled with (*R*,*R*)-*E*-**FcD⁺** 0.1 wt% in E7 (A) and (*S*)-*E*-**FcD⁺** 0.1 wt% in E7 (B). Thickness of the θ-cell: 30 µm (A), 20 µm (B). Counterclockwise rotation of the defect line (highlighted with red arrow) with respect to rubbing direction (dashed line) indicates left-handed (LH) twist of cholesteric mesophase while clockwise rotation indicates right-handed (RH) twist. From the angle between defect line and rubbing direction the helix pitch (p) is calculated and displayed in the figure. HTP((*R*,*R*)-*E*-**FcD⁺**) = -1.2 µm^{-1}; HTP((*S*,*S*)-*E*-**FcD⁺**) = 0.3 µm^{-1}.

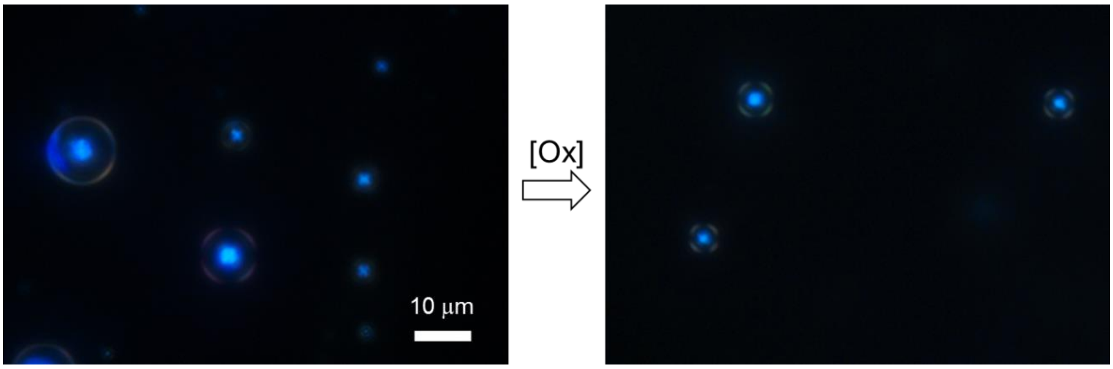

Figure S31. POM image (in reflection mode) of the LC droplets made of control mixture with short helix pitch ((*R*)-BB 6% in E7) before and after oxidation with $Fe(ClO_4)_3$ (25 mM, 5 min). It is clearly visible that treatment of droplets containing no redox active species with the oxidant does not lead to reflection color change (i.e. cholesteric helix pitch is constant).

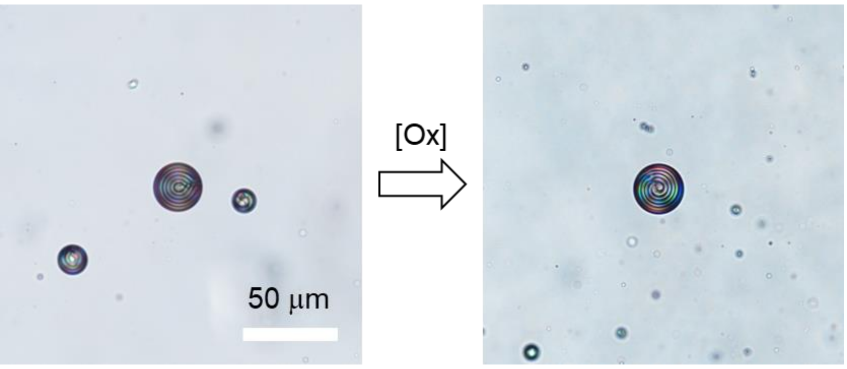

Figure S32. Optical image (in bright field) of the LC droplets made of control mixture ((*R*)-BB 0.5% in E7) before oxidation, after oxidation with $Fe(ClO_4)_3$ (25 mM, 30 min). The droplets containing no redox active chiral dopant do not show any visible changes of spiral optical pattern upon oxidation.

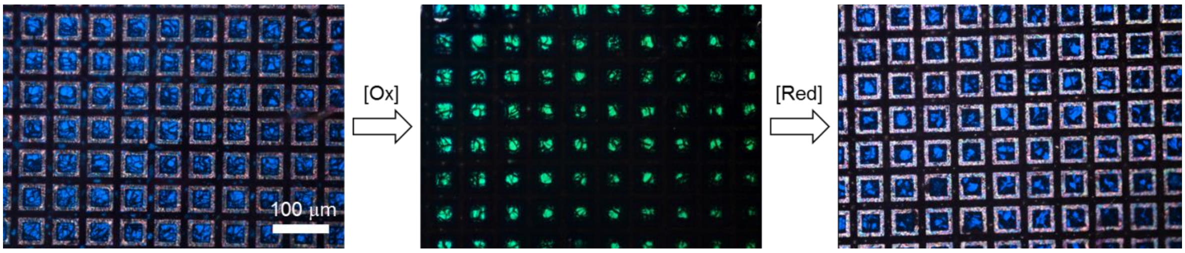

Figure S33. POM images (in reflection mode) of the LC layer ((*R,R*)-*E*-**FcD** 4 wt% +(*R*)-BB 5 wt% in E7) supported by copper grid subjected to oxidation and subsequent reduction with $Fe(ClO_4)_3$ (25 mM, 10 min) and ascorbic acid (50 mM, 10 min) as aqueous solutions, respectively.

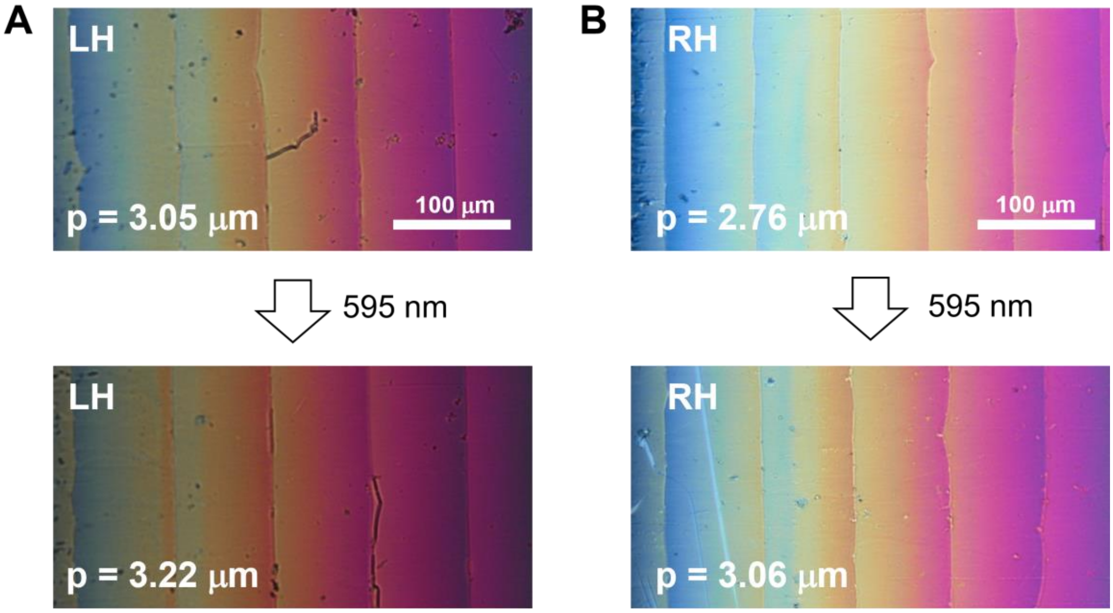

Figure S34. POM image of the wedge cell filled with thermally equilibrated (*R,R*)-**FcD** 2 wt% in E7 (A) and thermally equilibrated (*S,S*)-**FcD** 2 wt% in E7 (B) upon irradiation with 595 nm light.

The exposure of the cells to yellow light (595 nm, 1 h) initiates the partial isomerization form *E*-*syn*-**FcD** to *Z*-*anti*-**FcD**, leading to the unwinding of the cholesteric pitch. Keeping in mind that the HTP can be considered as sum of contributions of all chiral species present in the system and knowing distribution of *E* and *Z* states in the thermally equilibrated (*E*:*Z* ratio 67:33) and photochemical equilibrium (*E*:*Z* ratio 8020) it was possible to estimate the HTP of the pure Z-anti state, which were calculated as -21.6 μm^{-1} and 27.5 μm^{-1} for (*R*,*R*)-**FcD** and (*S*,*S*)-**FcD**, respectively. Wedge angle of the cell: tanθ=0.0192. LH and RH correspond to left- and right-handed cholesteric structure, respectively; p - cholesteric pitch.

9. HPLC Chromatograms

<Chromatogram>

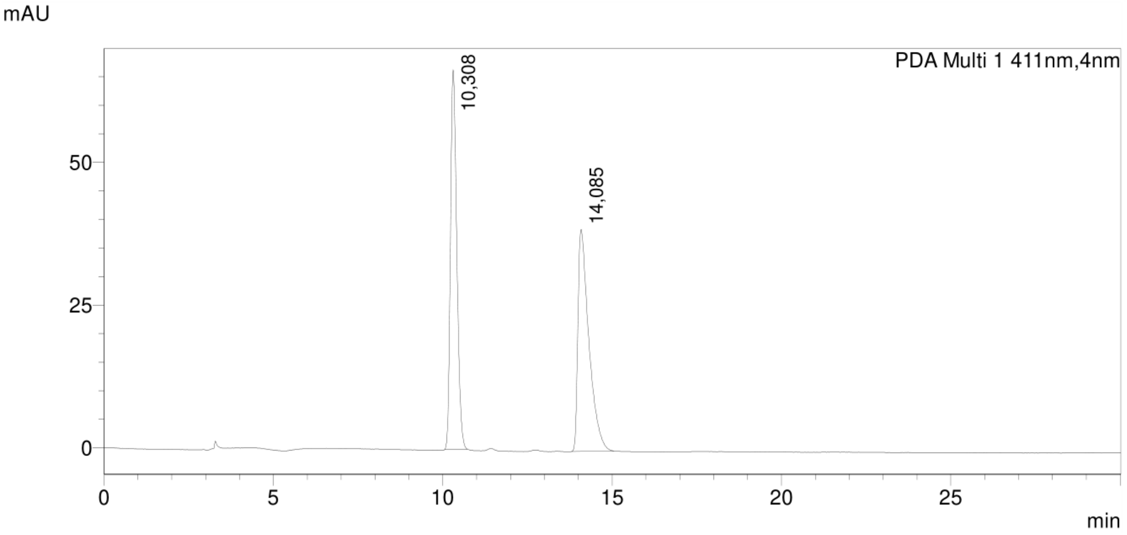

<Peak Table>

PDA Ch1 411nm

Peak#	Ret. Time	Area%
1	10,308	49,788
2	14,085	50,212
Total		100,000

HPLC chromatogram of (*rac*)-**FcKetone** (Chiralcel OD-H, *n*-heptane/2-propanol 97:3, 1.0 mL/min).

<Chromatogram>

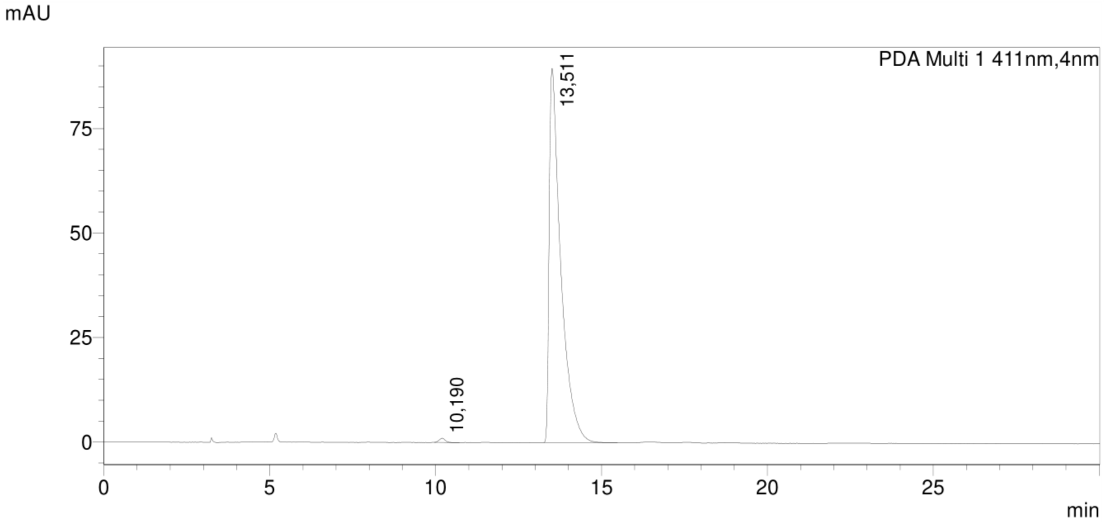

<Peak Table>

PDA Ch1 411nm

Peak#	Ret. Time	Area%
1	10,190	0,567
2	13,511	99,433
Total		100,000

HPLC chromatogram of (*R*)-**FcKetone** (Chiralcel OD-H, *n*-heptane/2-propanol 97:3, 1.0 mL/min).

<Chromatogram>

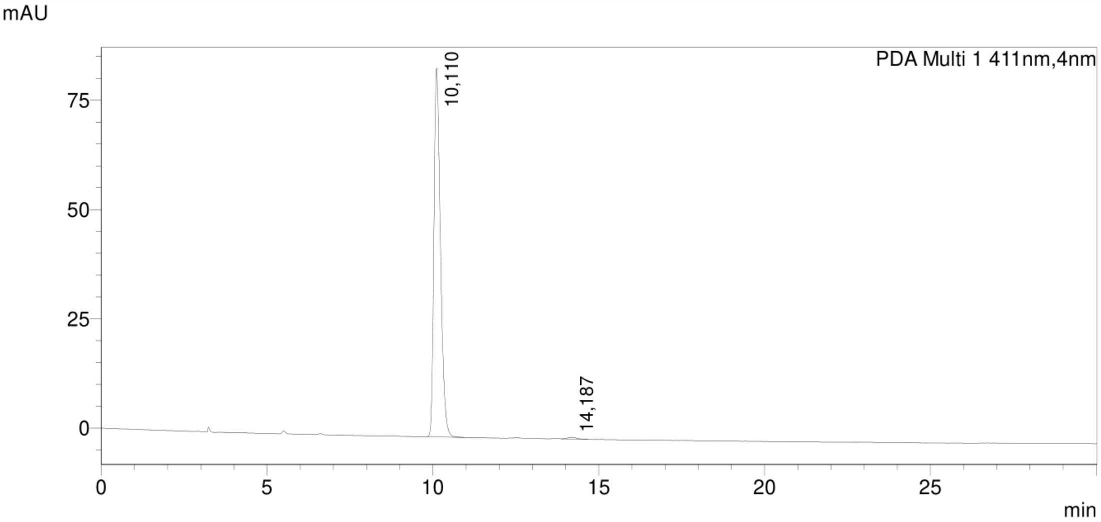

<Peak Table>

PDA Ch1 411nm

Peak#	Ret. Time	Area%
1	10,110	99,372
2	14,187	0,628
Total		100,000

HPLC chromatogram of (*S*)-**FcKetone** (Chiralcel OD-H, *n*-heptane/2-propanol 97:3, 1.0 mL/min).

<Chromatogram>

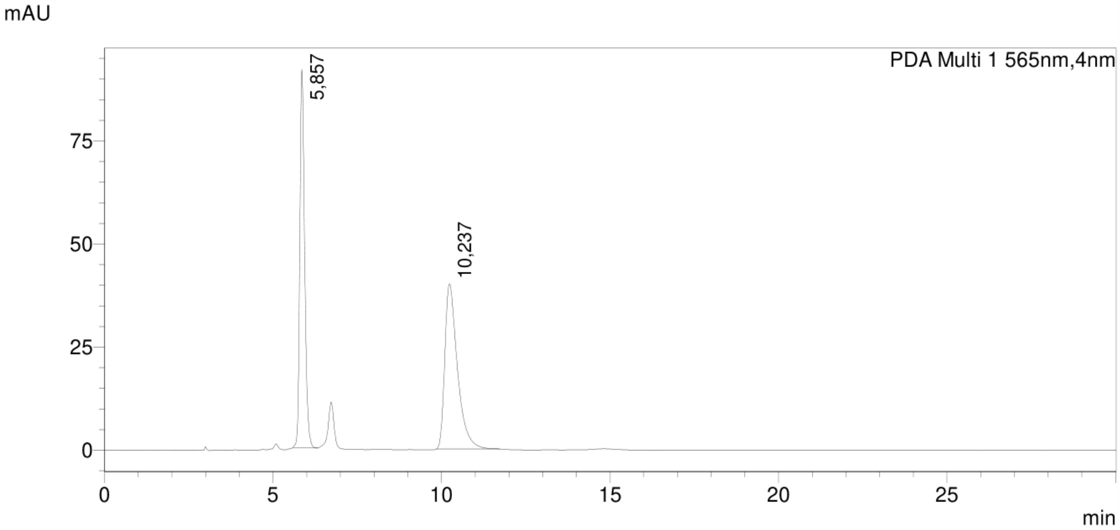

<Peak Table>

PDA Ch1 565nm

Peak#	Ret. Time	Area%
1	5,857	46,736
2	10,237	53,264
Total		100,000

HPLC chromatogram of (*rac*)-**FcD** (Chiralcel OD-H, *n*-heptane/2-propanol 95:5, 1.0 mL/min).

<Chromatogram>

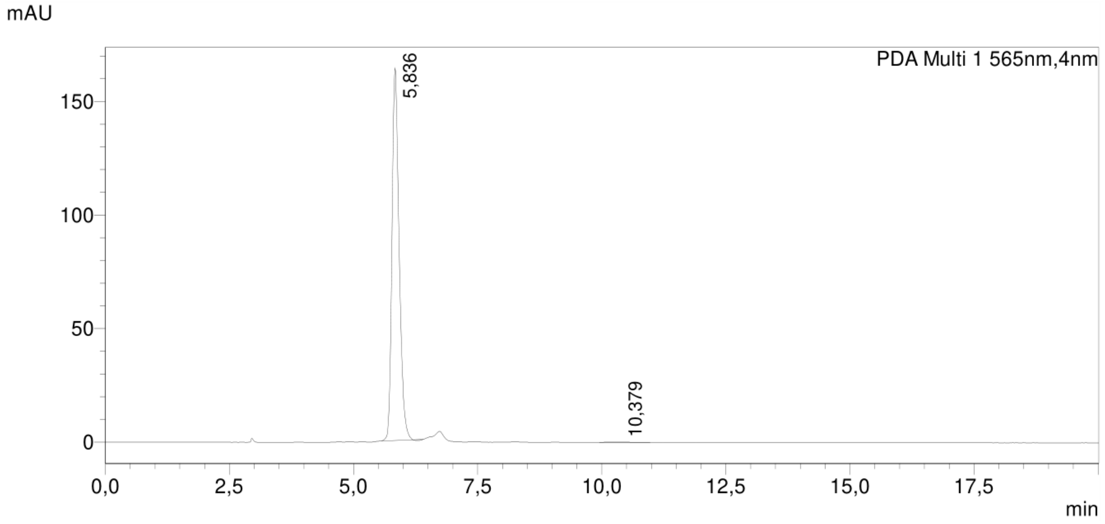

<Peak Table>

PDA Ch1 565nm

Peak#	Ret. Time	Area%
1	5,836	99,700
2	10,379	0,300
Total		100,000

HPLC chromatogram of (*R*,*R*)-**FcD** (Chiralcel OD-H, *n*-heptane/2-propanol 95:5, 1.0 mL/min).

<Chromatogram>

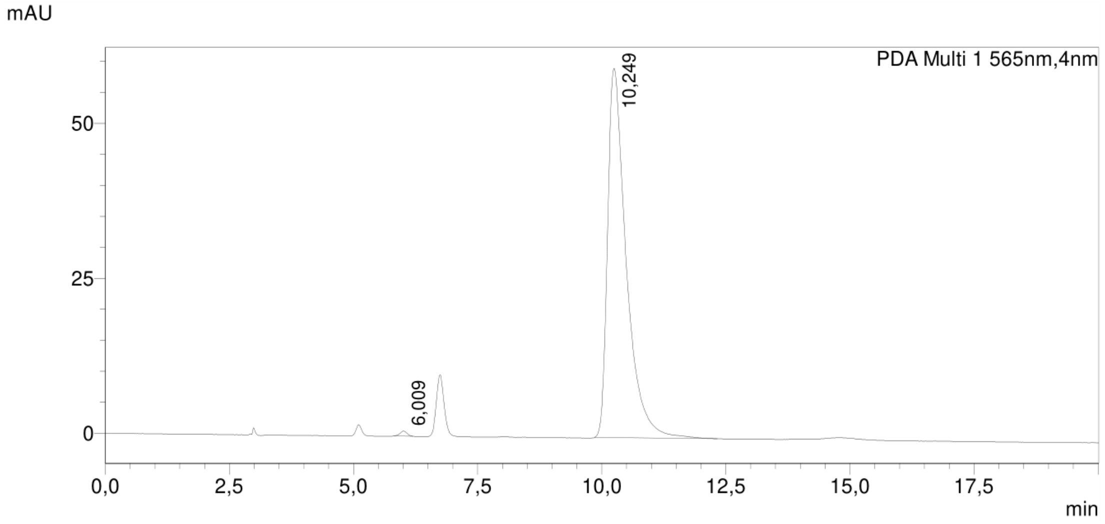

<Peak Table>

PDA Ch1 565nm

Peak#	Ret. Time	Area%
1	6,009	0,520
2	10,249	99,480
Total		100,000

HPLC chromatogram of (*S*,*S*)-**FcD** (Chiralcel OD-H, *n*-heptane/2-propanol 95:5, 1.0 mL/min).

10. NMR Spectra of New Compounds

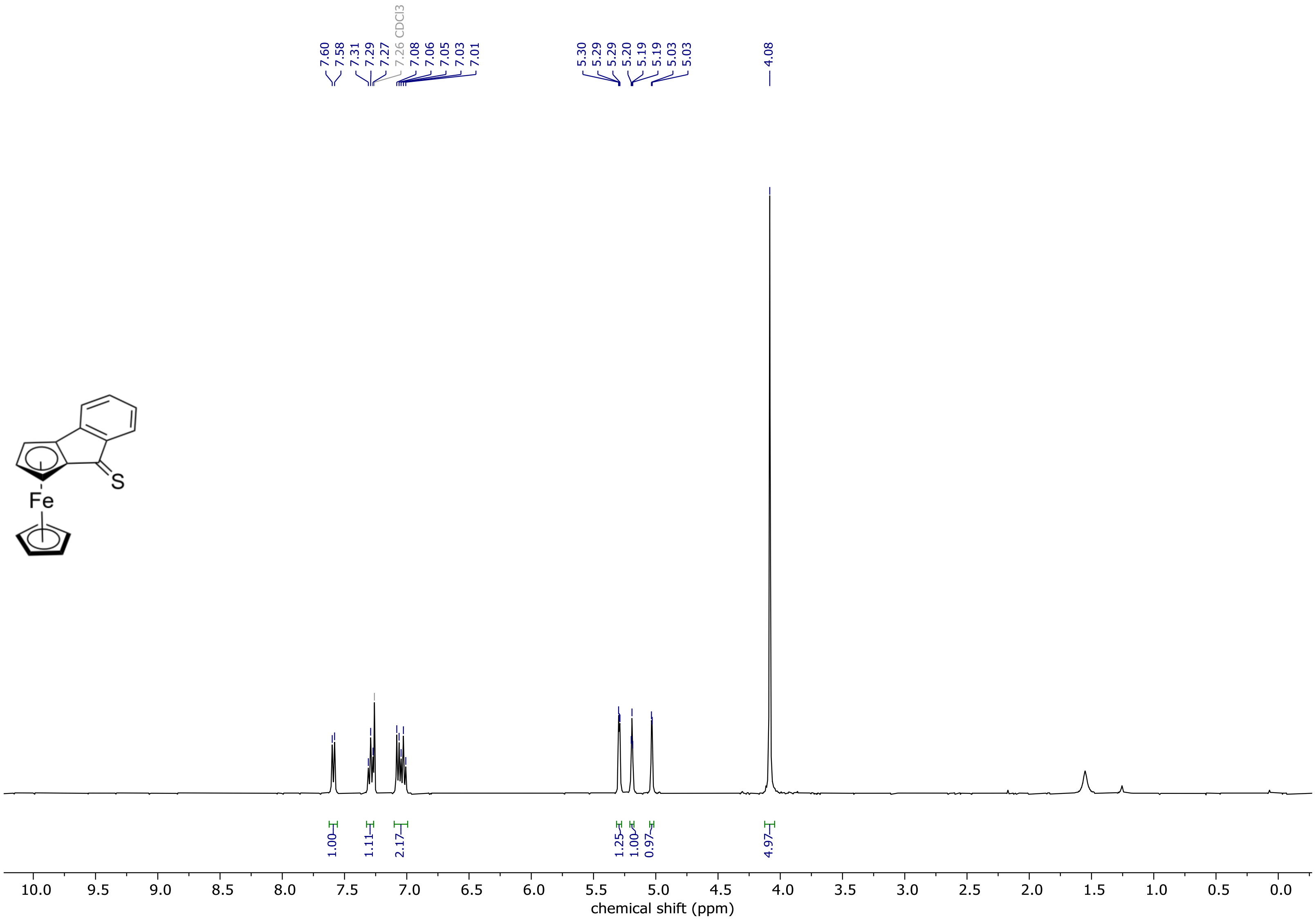

^{1}H-NMR spectrum of compound (*S*)-**FcThioketone** (400 MHz, $CDCl_3$, 25 °C).

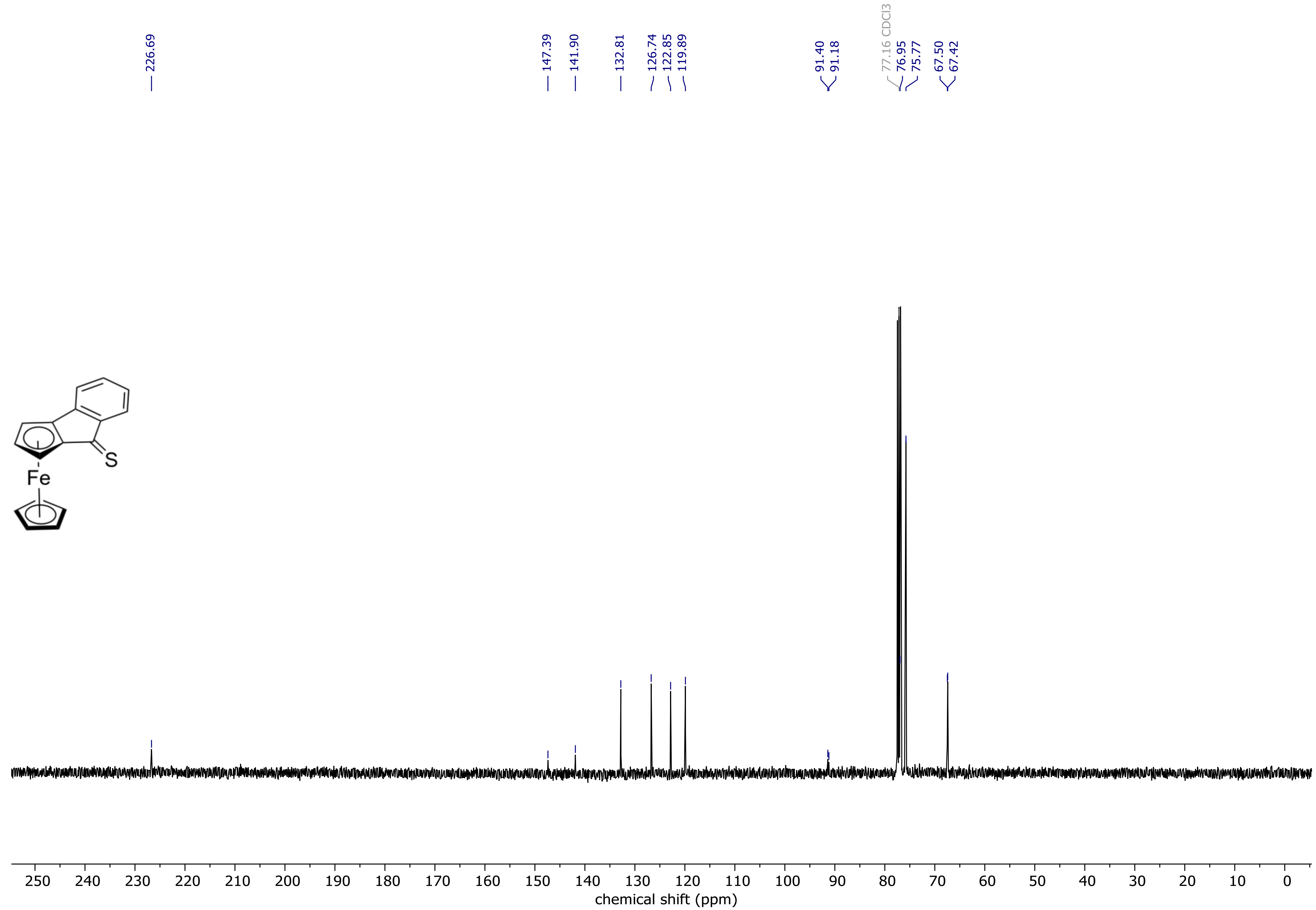

$^{13}C\{^{1}H\}$-NMR spectrum of compound (*S*)-**FcThioketone** (101 MHz, $CDCl_3$, 25 °C).

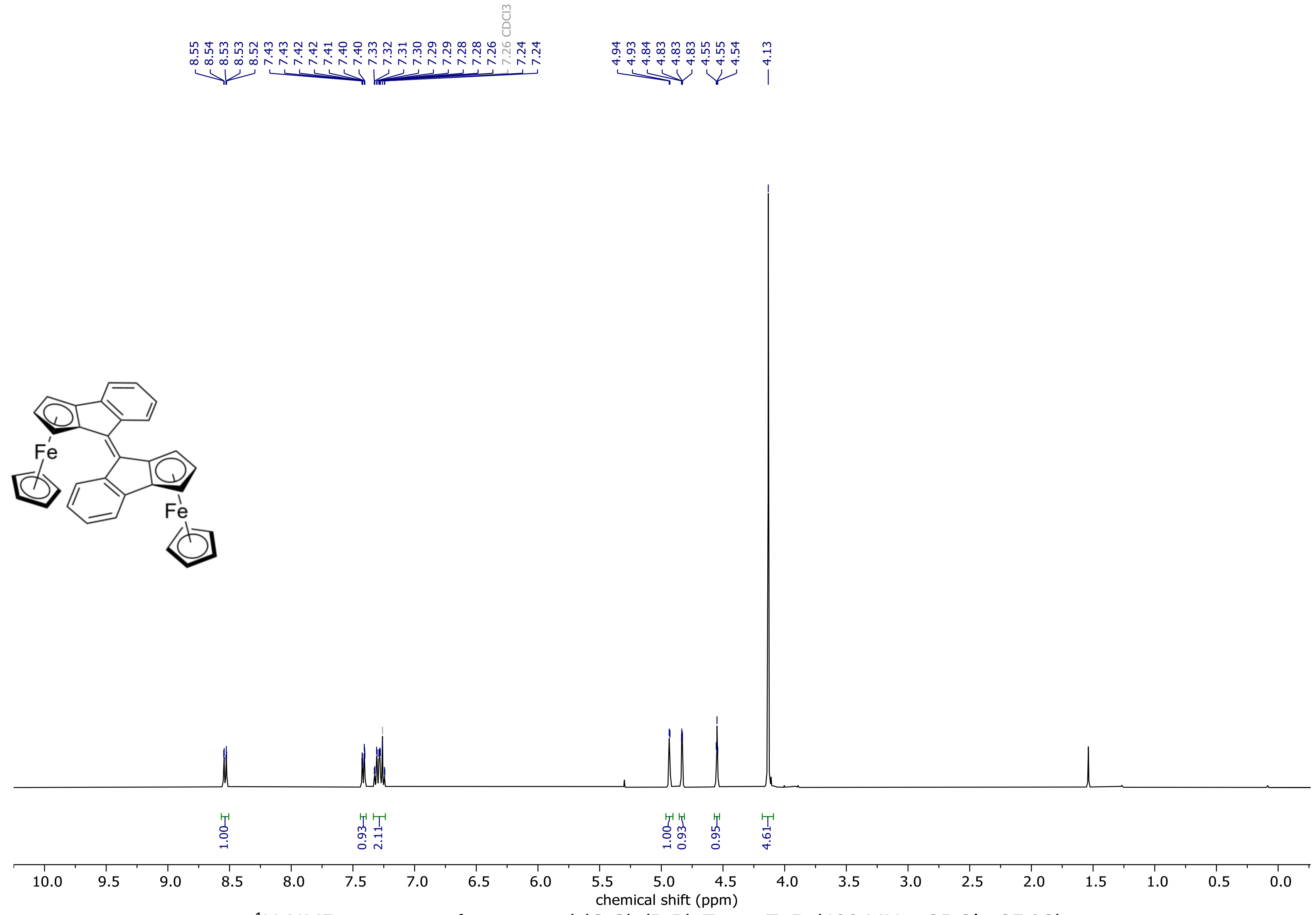

^{1}H-NMR spectrum of compound (*S*,*S*)-(*P*,*P*)-*E*-*syn*-**FcD** (400 MHz, $CDCl_3$, 25 °C).

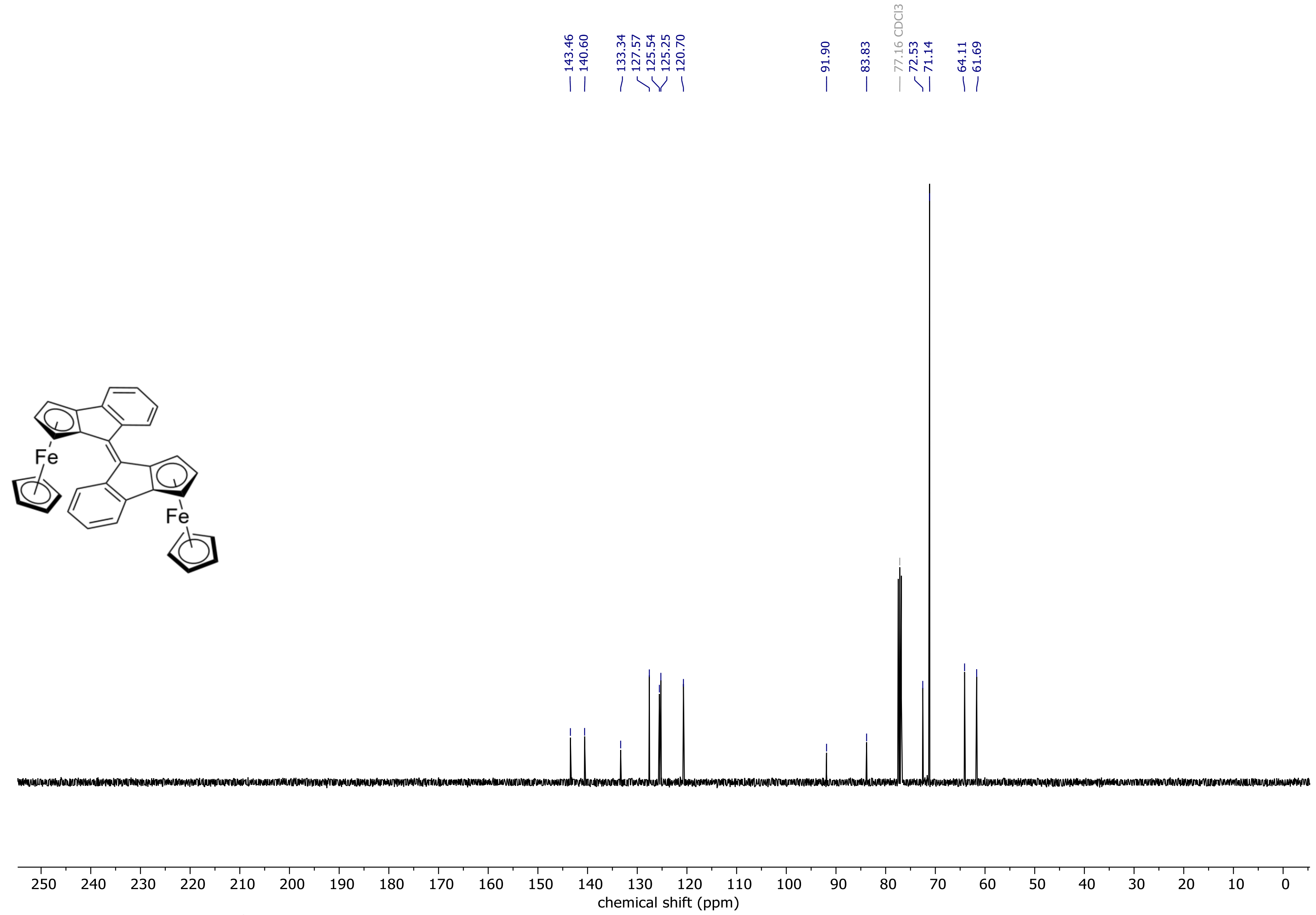

$^{13}C\{^{1}H\}$-NMR spectrum of compound (*S*,*S*)-(*P*,*P*)-*E*-*syn*-**FcD** (101 MHz, $CDCl_3$, 25 °C).

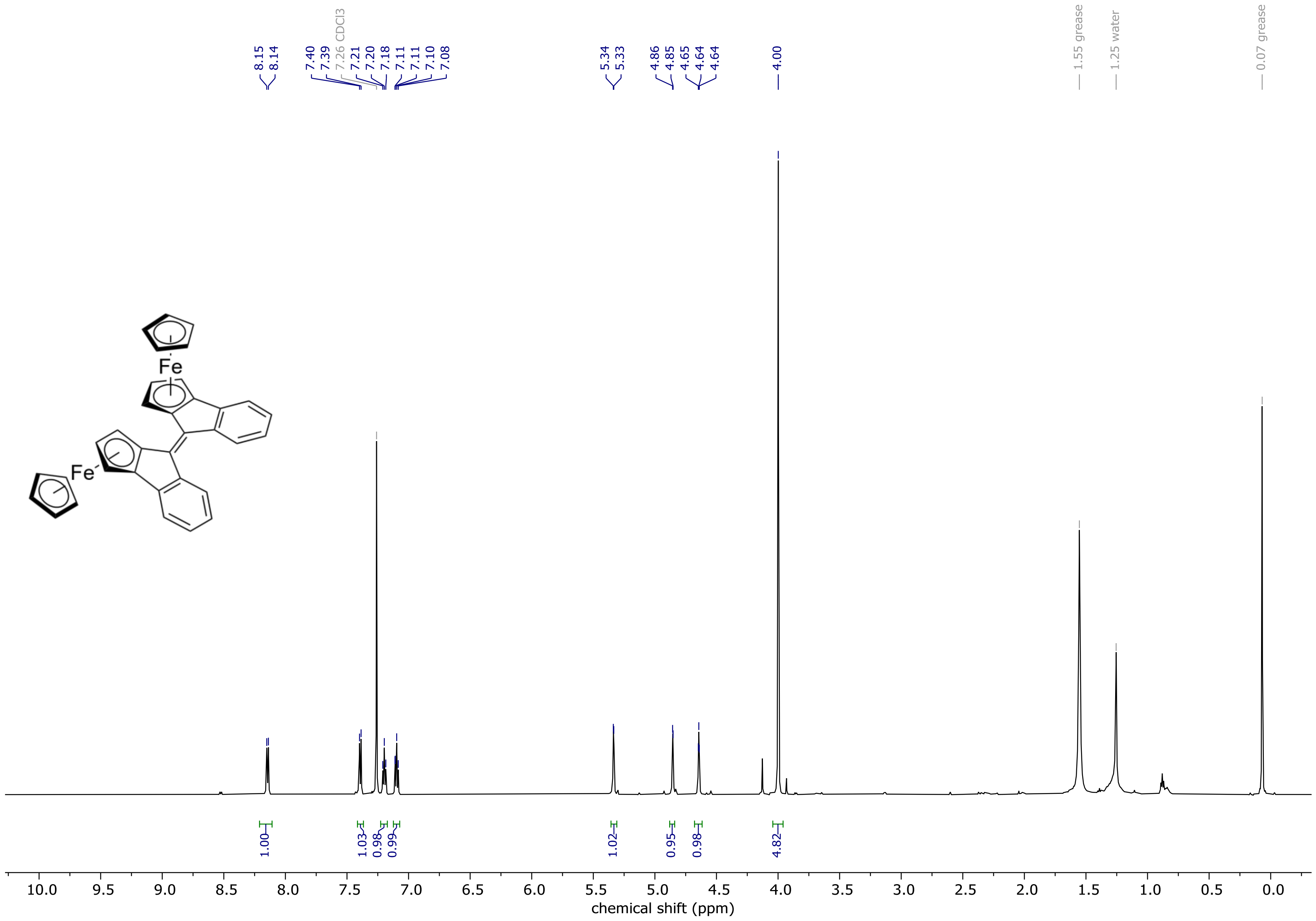

^{1}H-NMR spectrum of compound (*R*,*R*)-(*M*,*M*)-*Z*-*anti*-**FcD** (600 MHz, $CDCl_3$, 25 °C).

11. References

[1] R. Deng, Y. Huang, X. Ma, G. Li, R. Zhu, B. Wang, Y.-B. Kang, Z. Gu, *J. Am. Chem. Soc.* **2014**, *136*, 4472–4475.
[2] D.-W. Gao, Q. Yin, Q. Gu, S.-L. You, *J. Am. Chem. Soc.* **2014**, *136*, 4841–4844.
[3] S. Grimme, *J. Chem. Theory Comput.* **2019**, *15*, 2847–2862.
[4] P. Pracht, F. Bohle, S. Grimme, *Phys. Chem. Chem. Phys.* **2020**, *22*, 7169–7192.
[5] P. Pracht, S. Grimme, C. Bannwarth, F. Bohle, S. Ehlert, G. Feldmann, J. Gorges, M. Müller, T. Neudecker, C. Plett, S. Spicher, P. Steinbach, P. A. Wesołowski, F. Zeller, *J. Chem. Phys.* **2024**, *160*, 114110.
[6] C. Bannwarth, S. Ehlert, S. Grimme, *J. Chem. Theory Comput.* **2019**, *15*, 1652–1671.
[7] F. Neese, F. Wennmohs, U. Becker, C. Riplinger, *J. Chem. Phys.* **2020**, *152*, 224108.
[8] S. Grimme, A. Hansen, S. Ehlert, J.-M. Mewes, *J. Chem. Phys.* **2021**, *154*, 064103.
[9] V. Barone, M. Cossi, *J. Phys. Chem. A* **1998**, *102*, 1995–2001.
[10] L. Krause, R. Herbst-Irmer, G. M. Sheldrick, D. Stalke, *J. Appl. Cryst.* **2015**, *48*, 3–10.
[11] G. M. Sheldrick, *Acta Cryst. A* **2015**, *71*, 3–8.
[12] G. M. Sheldrick, *Acta Cryst. A* **2008**, *64*, 112–122.
[13] O. V. Dolomanov, L. J. Bourhis, R. J. Gildea, J. A. K. Howard, H. Puschmann, *J. Appl. Cryst.* **2009**, *42*, 339–341.
[14] M. Stalder, M. Schadt, *Opt. Lett.* **1996**, *21*, 1948.
[15] D. Kasyanyuk, K. Slyusarenko, J. West, M. Vasnetsov, Y. Reznikov, *Phys. Rev. E* **2014**, *89*, 022503.
[16] A. Ryabchun, R. Jamagne, J. Echavarren, M. Patanapongpibul, L. Zhang, N. Katsonis, D. A. Leigh, *Chem* **2024**, https://doi.org/10.1016/j.chempr.2024.03.013
[17] T. Orlova, S. J. Aßhoff, T. Yamaguchi, N. Katsonis, E. Brasselet, *Nat. Commun.* **2015**, *6*, 7603.
